# Multi-Omics Analysis Identifies TTYH3 and MPG as Candidate Osteoporosis-Associated Biomarkers in Osteoblasts and Characterizes Spatial Heterogeneity of SPP1 in the Osteoporotic Bone Microenvironment

**DOI:** 10.3390/biomedicines14092126

**Published:** 2026-09-20

**Authors:** Hengyi Diao, Qianning Li, Yucheng Tu, Yang Wu, Qiaojun Huang, Fangang Meng, Weishen Chen

**Affiliations:** 1 Department of Joint Surgery, The First Affiliated Hospital of Sun Yat-sen University, Guangzhou 510080, China; diaohy@mail2.sysu.edu.cn (H.D.);; 2 Laboratory of General Surgery, The First Affiliated Hospital of Sun Yat-sen University, Guangzhou 510080, China; 3 Department of Sports Medicine, The First Affiliated Hospital of Sun Yat-sen University, Guangzhou 510080, China

**Keywords:** osteoporosis, osteoblasts, ScRNA-seq, bulk RNA-seq, spatial transcriptome sequencing

## Abstract

**Background**: Osteoporosis (OP) arises from dysregulated bone metabolism driven by genetic and epigenetic factors. Osteoblasts are central to bone formation, and their functional heterogeneity—shaped by genetic background and receptor expression profiles—may critically influence OP susceptibility. This study aimed to identify osteoblast-specific genes robustly associated with OP and elucidate their underlying pathogenic mechanisms. **Methods**: We analyzed single-cell RNA sequencing (scRNA-seq), bulk RNA sequencing (bulk RNA-seq), and spatial transcriptomics (ST) datasets of osteoblasts. Differentially expressed genes (DEGs) were identified from scRNA-seq and bulk RNA-seq datasets, followed by Gene Ontology (GO) and Kyoto Encyclopedia of Genes and Genomes (KEGG) analysis. Three machine learning methods and an artificial neural network (ANN)-based weighting analysis, together with weighted gene co-expression network analysis (WGCNA), were used to prioritize candidate OP-associated genes. ST data were integrated with CellChat analysis based on scRNA-seq data to investigate spatial expression patterns and potential cell–cell communication. Candidate genes were validated by immunohistochemical staining in human femoral head samples and quantitative real-time PCR (qRT-PCR) in MC3T3-E1 cells. **Results**: The intersecting DEGs from the scRNA-seq and bulk RNA-seq datasets were potentially related to inhibition of ossification. Three machine learning methods identified five osteoporosis-associated candidate genes: TTYH3, NRBP2, MPG, HSPG2, and GPR153, which were subsequently evaluated using ANN-based weighting analysis. Among these, TTYH3 and MPG were identified as OP-related genes by WGCNA. Integration of the scRNA-seq, ST, and CellChat results suggested that SPP1 was highly expressed in osteoblasts from the OP sample and exhibited a spatially heterogeneous expression pattern. Immunohistochemical staining of human femoral head tissues from individuals with normal bone mass and osteoporosis, together with qRT-PCR analysis in MC3T3-E1 cells, further validated the differential expression of TTYH3 and MPG. **Conclusions**: This study identified candidate osteoporosis-associated biomarkers and provided insights into potential pathogenic mechanisms, thereby establishing a basis for future mechanistic and clinical validation.

## 1. Introduction

The global aging population and the rising prevalence of osteoporosis (OP) have converged into a major public health challenge, posing substantial threats to skeletal health and driving escalating healthcare expenditures, including costs for pharmacotherapy, diagnostic imaging, and surgical intervention in OP patients [1,2,3]. Without timely and appropriate management, OP inevitably progresses to fragility fractures. Globally, OP accounts for approximately 8.9 million fractures annually; among affected individuals, only 20% regain pre-fracture functional status, 50% develop long-term disability, and 20% die within one year of fracture [2,4]. Projections from a China-specific epidemiological model indicate that the incidence of OP-related fractures will double by 2035, with associated direct medical costs projected to reach ¥132 billion, imposing a profound socioeconomic burden on individuals, families, and healthcare systems [5].

Bone tissue is a highly dynamic, cellularly heterogeneous organ composed of multiple functionally specialized cell types, including osteocytes, osteoblasts, osteoclasts, fibroblasts, mesenchymal stromal cells (MSCs), and hematopoietic stem cells (HSCs). Osteoblasts, differentiated from MSCs, are the principal bone-forming cells responsible for synthesizing, secreting, and orchestrating the deposition of the organic bone matrix, predominantly type I collagen, and regulating its subsequent mineralization. Critically, osteoblasts also coordinate bone remodeling by modulating osteoclast activity through RANKL/OPG signaling, thereby maintaining physiological equilibrium between bone formation and resorption [6,7]. During bone remodeling, osteoclasts resorb aged or damaged bone tissue, whereas osteoblasts subsequently synthesize and mineralize new bone matrix to fill the resorption lacunae, thereby restoring skeletal integrity [8]. Dysfunctional osteoblasts impair extracellular matrix synthesis and mineralization—key defects underlying aberrant bone remodeling in osteoporosis. Consequently, pharmacological enhancement of osteoblast activity represents a cornerstone therapeutic strategy, aimed at stimulating de novo bone formation, restoring skeletal homeostasis, and improving both bone mass and microarchitectural integrity [9,10].

Single-cell RNA sequencing (scRNA-seq) enables high-resolution, cell-type-specific transcriptomic profiling, and has been extensively applied across diverse biomedical domains—including oncology, stem cell biology, and cardiovascular and respiratory diseases [11,12,13]. ScRNA-seq enables transcriptomic profiling at single-cell resolution, thereby facilitating the characterization of cellular and molecular heterogeneity, the identification of disease-associated marker genes and rare pathogenic cell populations, and the elucidation of underlying genetic and molecular disease mechanisms [14,15].

Spatial transcriptomics (ST) is an emerging high-throughput technology that enables spatially resolved transcriptomic profiling—capturing gene expression patterns while preserving the native tissue architecture and positional information [16]. By integrating transcriptomic profiles with spatial coordinates, ST enables the systematic investigation of cellular heterogeneity, tissue architecture, and molecular interactions within native tissue microenvironments. In contrast to bulk RNA-seq and scRNA-seq, which dissociate cells from their anatomical context, ST provides spatially resolved gene expression maps, delineates functional cellular niches, and identifies disease-associated transcriptional programs with anatomical precision [17,18]. Since its introduction, spatial transcriptomics has been widely applied in oncology, neuroscience, immunology, and regenerative medicine, yielding mechanistic insights into disease progression, tissue regeneration, and immune cell organization within native tissue contexts [19].

We analyzed scRNA-seq, bulk RNA-seq, and ST of osteoblasts from the GEO database. Integrated bioinformatics approaches, including DEGs, WGCNA, machine learning, and CellChat analysis, identified two candidate osteoporosis-associated biomarkers (TTYH3 and MPG) and one spatially variable gene (SPP1). Combined with clinical validation and experimental verification, these findings provide a basis for future mechanistic and clinical validation.

## 2. Materials and Methods

### 2.1. Data Collection

Four datasets (GSE147287, GSE156508, GSE284089, and GSE169396) were retrieved from the Gene Expression Omnibus (GEO) database (https://www.ncbi.nlm.nih.gov/geo/, accessed on 18 August 2026). The osteoporosis-related scRNA-seq dataset GSE147287 comprises femoral head tissue samples from two donors: a 67-year-old postmenopausal woman with osteoporosis (PMOP; sample GSM4423510) and an 84-year-old woman with primary osteoarthritis but preserved bone mineral density (sample GSM4423511). The publicly available scRNA-seq data included gene-barcode matrices, feature annotations, and UMI count matrices. No additional ethical approval was required as all data were de-identified and obtained under IRB-approved protocols. GSE156508 (GPL16686 platform) contains bulk RNA-seq profiles of primary human osteoblasts isolated from trabecular bone explants obtained during hip replacement surgery, comprising six osteoarthritis patients with normal bone mineral density (GSM4732740–GSM4732745) and six osteoporosis patients with documented fragility fractures (GSM4732746–GSM4732751).

### 2.2. Quality Control and the Dimensionality Reduction

Gene expression data were imported into the R v4.4.3 and Seurat (version 5.5.0) using the Read10X() function [20]. For the GSE147287 scRNA-seq dataset, low-quality cells (<300 or >7000 transcripts per cell, >10% mitochondrial genes, or >3% hemoglobin genes) and genes detected in fewer than three cells were excluded. Following quality control, the two samples were integrated and normalized, followed by the selection of highly variable genes and principal component analysis (PCA). Batch effects were corrected using Harmony. Principal components for downstream analysis were selected based on JackStraw analysis and elbow plots, and PCs 1–15 were used for graph-based clustering. Cell clusters were identified using Seurat’s FindNeighbors and FindClusters functions and visualized using UMAP and t-SNE [21].

### 2.3. Annotation and DEGs Identification from Cell-Clustering

Cell clusters were annotated through manual curation guided by canonical marker genes reported in the literature, and annotation consistency was further cross-checked and refined using SingleR. Differentially expressed genes (DEGs) between the osteoporosis and osteoarthritis groups were identified within the cell clusters of interest using the Wilcoxon rank-sum test implemented in Seurat’s FindMarkers function. Cluster-associated DEGs were defined as genes meeting all of the following criteria: expression in at least 25% of cells within the target cluster (min.pct = 0.25), an adjusted *p* value < 0.05, and |avg_log2FC| > 0.5.

### 2.4. Bulk RNA-Seq Dataset Analysis

The expression matrix of the bulk RNA-seq dataset GSE156508 was analyzed in R using the “limma” package (version 3.60.6). The quality control was conducted by keeping the genes that have an expression level greater than 1 in at least 20% of the samples filter out low-expression genes. Based on the construction of a matrix for fracture and a matrix for osteoarthritis and for comparison. Using lmFit to fit the linear model, makeContrasts to define the comparison groups, and finally adjust the variance part of the *t*-test using empirical Bayes method. Based on the threshold of *p* value < 0.05 and |logFC| > 0.25, the differentially expressed genes (DEGs) were selected.

### 2.5. WGCNA for Candidate Genes

In this study, the R package “WGCNA” (version 1.73) was utilized to filter the combined data for outliers and then construct a co-expression network. Highly similar genes were grouped into co-expression modules based on correlation patterns, with soft-thresholding power (β) selected to optimize network robustness. A topological overlap matrix (TOM) was generated from the weighted gene correlation matrix and used for hierarchical clustering and module detection via the Dynamic Tree Cut algorithm. Module eigengenes (MEs), defined as the first principal component of each module’s expression matrix, were calculated to represent module expression profiles [22]. The chosen modules were analyzed using WGCNA and important module genes were found for further investigation. Modules associated with OP were selected for subsequent candidate-gene screening.

### 2.6. Identification and Functional Enrichment Analysis of Intersecting DEGs

In this study, intersecting DEGs were identified from the bulk RNA-seq and scRNA-seq datasets using the R package “VennDiagram” (version 1.7.3). To investigate the biological functions of these intersecting DEGs, Gene Ontology (GO) and Kyoto Encyclopedia of Genes and Genomes (KEGG) pathway enrichment analyses were performed using the R package “clusterProfiler” (version 4.12.6). Significantly enriched GO terms and KEGG pathways were visualized as bar plots and dot plots using the R package “enrichplot” (version 1.24.4) [23].

### 2.7. Candidate Gene Prioritization Using Machine Learning Techniques

To identify candidate genes associated with osteoporosis in the intersecting DEGs, we utilized the R package “glmnet” (version 4.1.10) to implement the Least Absolute Shrinkage and Selection Operator (LASSO) algorithm. Additionally, SVM-RFE and Random Forest were applied as complementary candidate-gene prioritization methods, and the genes were ranked according to method-specific metrics. An artificial neural network (ANN)-based weighting analysis was subsequently performed using the R packages “neuralnet” (version 1.44.2) and “NeuralNetTools” (version 1.5.3) to descriptively rank the relative contributions of the preselected genes.

### 2.8. Subclusters and Pseudotime Trajectory Analysis

Selected cell clusters were extracted and subjected to subcluster analysis using the standard scRNA-seq workflow. The “Monocle2” package (version 2.32.0) and “Monocle 3” package (version 1.3.7) was used to infer cell differentiation trajectories within the selected subclusters. The trajectories were visualized using the plot_cell_trajectory function [24].

### 2.9. Inference of Cell–Cell Interaction

Cell–cell interaction analysis was performed using the R package “CellChat” (version 2.1.2) based on known ligand–receptor interactions among cell clusters [25]. This algorithm computes ligand–receptor interaction probabilities to quantify signaling activity across communication pathways. CellChat analysis was conducted using the standard workflow, including the computeCommunProb, computeCommunProbPathway, and aggregateNet functions, with default parameters and a fixed randomization seed. The analysis focused on “Secreted Signaling” pathways and used the precompiled human protein–protein interaction (PPI) database as prior network knowledge. Finally, the ligand–receptor interactions supported by permutation testing (*p* < 0.05) were retained.

### 2.10. Spatial Transcriptomic Profiling

The spatial transcriptomic dataset GSE284089 comprised a single formalin-fixed, paraffin-embedded femoral head section (GSM8677818) from a 42-year-old male patient undergoing hip replacement. GSE284089 contained spatial and single-cell transcriptomic profiles of human femoral head bone tissue generated using the Visium CytAssist Spatial Gene Expression platform. The aggregated Hierarchical Data Format version 5 (HDF5) expression matrix was imported into R and processed using R v4.4.3 and Seurat (version 5.5.0) for quality control, normalization, batch correction, dimensionality reduction, and clustering analysis. Low-quality spatial spots with fewer than 200 detected features were removed, followed by SCTransform normalization. Transcriptionally distinct spatial clusters were identified through dimensionality reduction and Louvain-based graph clustering [16]. Cluster marker genes were identified using the two-sided Wilcoxon rank-sum test implemented in Seurat with Bonferroni correction, using thresholds of |avg_log2FC| > 1 and adjusted *p* value < 0.05. Spatially variable genes (SVGs) were identified among the variable features of the SCT assay using Seurat’s FindSpatiallyVariableFeatures function with Moran’s I.

### 2.11. Cell Type Annotation, Deconvolution and Spatial Mapping

The scRNA-seq dataset GSE169396 contained transcriptomic profiles of 26,574 primary human femoral head cells isolated from three patients with osteoporosis and one patient with non-osteoporotic osteoarthritis. The GSE169396 scRNA-seq dataset was used as the reference atlas, whereas the spatial transcriptomic dataset served as the query dataset. Cell-type annotation transfer and spatial cell-type deconvolution were performed using the R package “SPOTlight” (version 1.16.0), which applies a seeded non-negative matrix factorization (NMF)-based approach to estimate cell-type composition within individual spatial spots. The inferred cell type proportions were subsequently incorporated into the Seurat object metadata for downstream spatial transcriptomic analyses [26].

### 2.12. GO, KEGG Enrichment Analysis and ssGSEA, GSVA

Functional enrichment analysis of cluster marker genes was performed using the “clusterProfiler” package. Gene symbols were converted to Entrez IDs using “org.Hs.eg.db”, followed by Gene Ontology (GO) and Kyoto Encyclopedia of Genes and Genomes (KEGG) enrichment analyses. Significantly enriched GO terms and KEGG pathways were visualized using bar and dot plots. Pathway activity was evaluated using Gene Set Variation Analysis (GSVA) and single-sample Gene Set Enrichment Analysis (ssGSEA) based on SCTransform-normalized spatial transcriptomic data. Custom gene sets in Gene Matrix Transposed (GMT) format were analyzed using the “GSEABase” and “GSVA” packages, and the resulting pathway scores were incorporated into the Seurat object metadata for downstream visualization using SpatialFeaturePlot, FeaturePlot, and VlnPlot.

### 2.13. Cell Trajectory Analysis

To characterize spatially resolved cellular transitions, trajectory analysis was performed on the spatial transcriptomic dataset using the Slingshot framework [27]. Following SCTransform normalization and principal component analysis (PCA) in Seurat, spatial spots were embedded in a low-dimensional space for trajectory inference. Slingshot was used to reconstruct developmental trajectories and calculate pseudotime values, which were subsequently projected onto spatial coordinates for visualization. Genes associated with pseudotime progression were identified using correlation analysis, and their dynamic expression patterns were visualized using spatial feature plots and pseudotime-dependent expression curves to delineate spatially organized transcriptional transitions [28].

### 2.14. Clinical Samples Obtaining

Clinical samples were obtained from patients undergoing total hip arthroplasty for clinical indications and were categorized into osteoporosis and normal bone mineral density (BMD) groups. Informed consent was obtained from all participants or their legal guardians before participation in the study. The samples consisted of surgically discarded femoral heads. Participants were categorized according to their preoperative quantitative computed tomography (qCT) T-scores: those with a T-score ≤ −2.5 were assigned to the osteoporosis group, whereas those with a T-score > −1 were assigned to the normal bone mass group. Because all tissues were collected during routine surgical procedures, sample collection imposed no additional burden on the patients.

### 2.15. Immunohistochemical Staining

The femoral head specimens were cut into tissue blocks and decalcified by immersion in ethylenediaminetetraacetic acid (EDTA) for 4–5 weeks, with the solution replaced weekly. Following decalcification, the samples were embedded in paraffin and sectioned. The resulting tissue sections were then deparaffinized and subjected to antigen retrieval. After removal of the storage solution, primary antibodies against TTYH3 (PA5-62800; Thermo Fisher Scientific, Waltham, MA, USA) and MPG (11481-2-AP; Proteintech, Wuhan, China) were applied, and the sections were incubated overnight in a humidified chamber at 4 °C. The sections were subsequently washed three times with phosphate-buffered saline (PBS) for 3 min each. An enzyme-labeled goat anti-mouse/rabbit IgG polymer was then applied, and the sections were incubated at 37 °C for 20 min. The sections were gently agitated and washed with PBS, followed by color development using 3,3′-diaminobenzidine (DAB). After rinsing with tap water to terminate color development, the sections were counterstained with hematoxylin for approximately 3 min and treated with ammonia water for bluing. Five distinct fields of view were selected at ×200 magnification, and the average integrated optical density (IOD) was calculated for each field.

### 2.16. Cell Culture and Osteogenic Differentiation

Mouse calvarial preosteoblastic (MC3T3-E1) Subclone 14 cells were purchased from Procell Life Science & Technology Co., Ltd. (Wuhan, China) and cultured in α-MEM supplemented with 6% fetal bovine serum (FBS) and 1% penicillin-streptomycin at 37 °C in a humidified atmosphere of 5% CO_2_. The α-MEM medium, fetal bovine serum, penicillin, and streptomycin were all purchased from HyClone (Logan, UT, USA). To induce osteogenic differentiation, MC3T3-E1 cells were cultured in an osteogenic induction medium (OIM, containing α-MEM, 10% fetal bovine serum, 10 mM β-glycerophosphate sodium and 50 μg/mL ascorbic acid). The control group was cultured in OIM alone without dexamethasone (DEX) treatment. For the DEX-treated group, a DEX-induced osteogenic dysfunction model was established by seeding MC3T3-E1 cells in 6-cm dishes at a density of 5 × 10^5^ cells per dish and treating them with 1 μM DEX in the same culture medium for 14 consecutive days. The induction medium was replaced every two days [29].

### 2.17. Mineralization Assay and Alizarin Red S Staining

Alizarin Red S staining was performed to assess matrix mineralization during osteogenic differentiation in both the control group and DEX-treated group. MC3T3-E1 cells were treated with or without DEX and were fixed in 4% paraformaldehyde for 30 min and stained with 1% Alizarin Red S (pH 4.2) for 10 min at room temperature. After washing with PBS to remove unbound dye, images were captured using an inverted microscope. For quantification, five different fields of view were selected and the average integrated optical density (IOD) was calculated for each field.

### 2.18. RNA Extraction and Quantitative Real-Time PCR (qRT-PCR) Analysis

Total RNA was isolated from cultured cells using TRIzol (Accurate Biotechnology (Hunan) Co., Ltd., Changsha, China) reagent. Complementary DNA (cDNA) was synthesized using Evo M-MLV RT Premix for qPCR (Accurate Biotechnology (Hunan) Co., Ltd., Changsha, China) with genomic DNA removal enzyme treatment. Quantitative real-time polymerase chain reaction (qRT-PCR) was performed using SYBR Green Pro Taq HS Premix (Accurate Biotechnology (Hunan) Co., Ltd., Changsha, China) on a 7500 Real-Time PCR System (Thermo Fisher Scientific, Waltham, MA, USA). Thermal cycling conditions were as follows: pre-denaturation at 95 °C for 30 s, followed by 40 cycles of denaturation at 95 °C for 5 s and annealing and extension at 60 °C for 30 s. β-actin was used as internal control, and the relative levels of TTYH3 and MPG were calculated using the 2^−ΔΔCt^ method [30]. The primer sequences were as follows: TTYH3 forward, 5′-CAGAGTGGGGAGGGGAGT-3′ and reverse, 5′-CTGGGCAGGTTGGCTGT-3′; MPG forward, 5′-GTCCTAGTCCGGCGACTTCC-3′ and reverse, 5′-CTTGTCTGGGCAGGCCCTTTGC-3′; β-actin forward, 5′-GGTGGCTTTTAGGATGGCAAG-3′ and reverse, 5′-ACTGGAACGGTGAAGGTGACAG-3′.

### 2.19. Statistical Analysis

**Figure 1:**
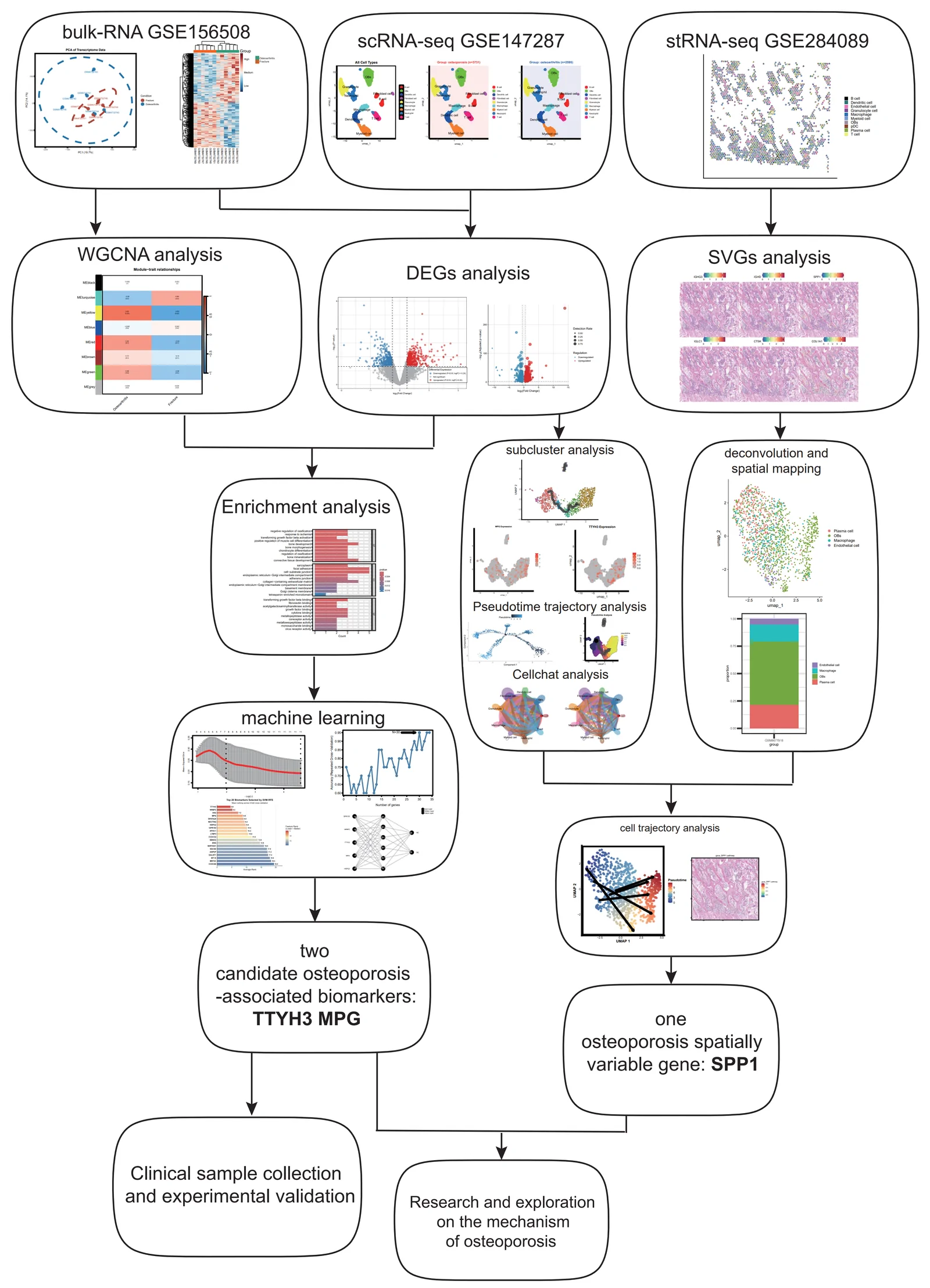
The flowchart of this study.

All statistical analyses were performed using R software v4.4.3 (https://www.r-project.org) and GraphPad Prism v10.3.1. Quantitative data are presented as mean ± standard deviation (SD). The distribution normality of continuous variables was assessed using the Shapiro–Wilk test. For comparisons between two groups, two-tailed Student’s *t*-test (paired or unpaired, as appropriate) was applied for normally distributed data. A two-sided *p* value < 0.05 was considered statistically significant. The research workflow is illustrated in Figure 1.

## 3. Results

### 3.1. Profiling scRNA-Seq Dataset of Osteoblasts in Osteoporosis

**Figure 2:**
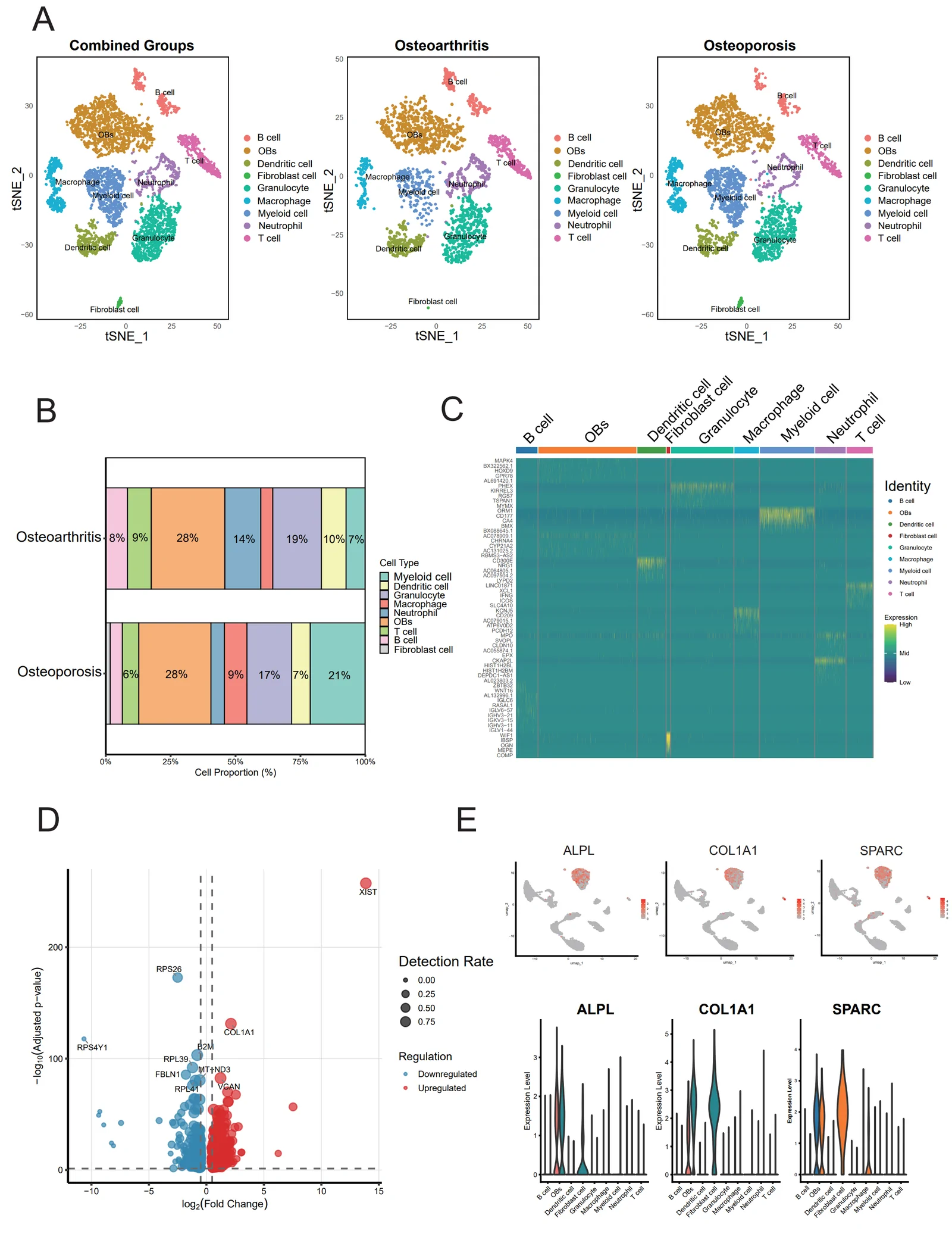
Analysis of scRNA-seq data: (**A**) T-SNE plots showing cell clustering patterns in the osteoporosis group, osteoarthritis group, and integrated dataset. (**B**) The distribution of cell types among groups. (**C**) Heatmap showing the top marker genes across the identified cell types. (**D**) Volcano plot displaying DEGs in the OBs cluster, highlighting the 10 most significant genes. (**E**) Expression patterns of ALPL, COL1A1, and SPARC across different cell clusters.

To identify candidate osteoporosis-associated biomarkers in osteoblasts, we analyzed scRNA-seq data from CD271+ (NGFR+) cells deposited in the Gene Expression Omnibus (GEO) database under accession number GSE147287. The dataset included one osteoporosis (OP) sample and one osteoarthritis (OA) sample and was used to investigate cellular diversity in femoral head tissue. Following initial quality control based on the predefined cell-filtering criteria, 3731 cells from the OP sample and 2595 cells from the OA sample were retained for transcriptomic profiling (Supplementary Figure S1A,B). After the identification of highly variable genes, the two samples were integrated, and principal component analysis (PCA) was performed using the highly variable genes across all cells (Supplementary Figure S1C,D). Fifteen principal components (PCs) and a clustering resolution of 0.08 were selected for downstream analysis, and Harmony was used to correct for batch effects between the samples (Supplementary Figure S1E). UMAP and t-SNE visualization initially identified 12 cell clusters. Clusters assigned to the same cell type based on their expression profiles and canonical marker genes were subsequently merged, resulting in nine major cell types: osteoblasts (OBs), B cells, dendritic cells, fibroblasts, granulocytes, neutrophils, myeloid cells, macrophages, and T cells (Figure 2A and Supplementary Figure S2A). All clusters were annotated based on their distinct expression profiles and previously reported marker genes. For example, clusters 0 and 3 were annotated as OBs based on the expression of ALPL, COL1A1, and SPARC (Figure 2E). Cluster 5 was annotated as a T-cell cluster based on the expression of PTPRC, CD3D, CD3E, CD4, and CD8A. Clusters 9 and 10 were annotated as B-cell clusters based on the expression of CD19, CD79A, and MS4A1. Cluster 11 was annotated as a fibroblast cluster based on the expression of ACTA2, PDGFRA, and PDGFRB (Supplementary Figure S2B). Differences in the proportions of the cell types between the two samples were calculated and visualized in a histogram (Figure 2B).

The top marker genes for each annotated cell type were visualized in a heatmap (Figure 2C). Subsequently, to examine differences in OBs between the OP and OA samples, we performed differential expression analysis after subsetting the OBs subcluster. Adjusted *p* values were calculated for all genes, and genes with an adjusted *p* value < 0.05 and |avg_log2FC| > 0.5 were considered differentially expressed genes (DEGs). We identified 1446 DEGs, comprising 1228 upregulated and 218 downregulated genes. XIST, RPS26, COL1A1, B2M, RPL39, FBLN1, VCAN, MT-ND3, RPL41, and RPS13 were the 10 DEGs showing the most statistically significant differences between the OP and OA samples in the OBs subcluster (Figure 2D).

### 3.2. Profiling Bulk RNA-Seq Dataset of Osteoblasts in Osteoporosis, WGCNA and Enrichment Analysis

**Figure 3:**
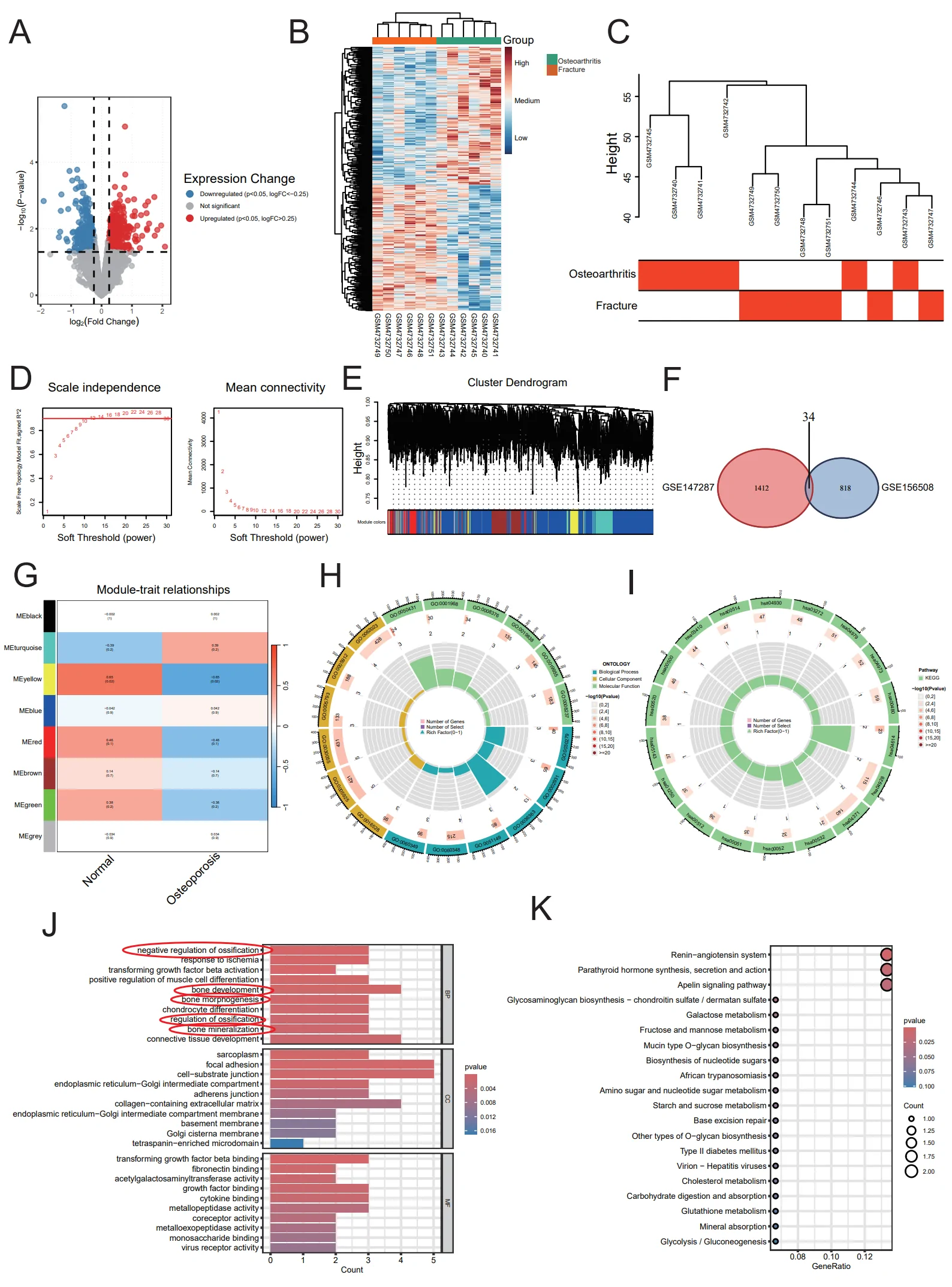
Analysis of bulk RNA-seq data, WGCNA, and enrichment analysis: (**A**) Volcano plot showing DEGs identified in GSE156508. (**B**) Heatmap illustrating differential gene expression patterns between the osteoarthritis and fracture groups. (**C**) Sample clustering dendrogram and trait heatmap depicting sample classification and trait relationships. (**D**) Selection of the optimal soft-thresholding power for scale-free network construction. (**E**) Gene dendrogram and module assignment. (**F**) Venn diagram showing overlapping DEGs between the two datasets. (**G**) Module–trait relationship heatmap identifying osteoporosis-associated modules. (**H**,**I**) Multi-layer circular plots displaying detailed GO and KEGG enrichment information. (**J**,**K**) GO and KEGG enrichment analyses of shared DEGs. The red circles highlight osteogenesis-related biological processes identified by GO enrichment analysis.

To investigate osteoporosis-associated DEGs in osteoblasts, we analyzed the bulk RNA-seq dataset GSE156508 from the GEO database, which included six OA samples from individuals with normal bone mass and six fracture samples from individuals with low bone mass. Using thresholds of *p* < 0.05 and |logFC| > 0.25, 852 DEGs were identified and visualized in a volcano plot (Figure 3A). The top 100 DEGs were further displayed in a heatmap to illustrate their expression patterns in the two groups (Figure 3B).

Given the high dimensionality of bulk RNA-seq data, weighted gene co-expression network analysis (WGCNA) was performed to identify osteoporosis-related gene modules. Sample clustering and trait correlation analysis demonstrated the overall distribution and relationships among samples (Figure 3C). A soft-threshold power of β = 9 was selected to establish a scale-free network, with a minimum module size of 10 (Figure 3D). Dynamic tree cutting and module merging identified eight co-expression modules (Figure 3E). Correlation analysis revealed that the black, turquoise, blue, and grey modules were positively associated with osteoporosis (Figure 3G), and these modules were selected as osteoporosis-related modules. Among them, the turquoise module exhibited highest positive correlation among the identified modules and its 5789 genes were identified as candidate genes for further analysis.

To further identify key DEGs associated with osteoporosis pathogenesis, we examined the intersecting DEGs between OBs in GSE147287 and osteoblasts in GSE156508. A total of 34 shared genes were identified, including 30 upregulated and 4 downregulated genes (Figure 3F). GO and KEGG enrichment analyses were subsequently performed to investigate their potential biological functions. GO analysis indicated that these DEGs were primarily enriched in biological processes related to “negative regulation of ossification,” “regulation of ossification,” “bone mineralization,” and “bone development”; cellular components associated with “focal adhesion” and “cell–substrate junction”; and molecular functions involving “transforming growth factor beta binding” and “growth factor binding.” KEGG analysis indicated significant enrichment in the “renin–angiotensin system,” “parathyroid hormone synthesis, secretion and action,” and “apelin signaling pathway” (Figure 3J,K). The integrated GO and KEGG enrichment visualizations further illustrated gene distributions, enrichment factors, and pathway contributions (Figure 3H,I). These findings suggest that the shared DEGs may participate in osteoporosis progression by regulating ossification, bone formation, and bone mass maintenance.

### 3.3. Machine Learning from the Differentially Expressed Genes

**Figure 4:**
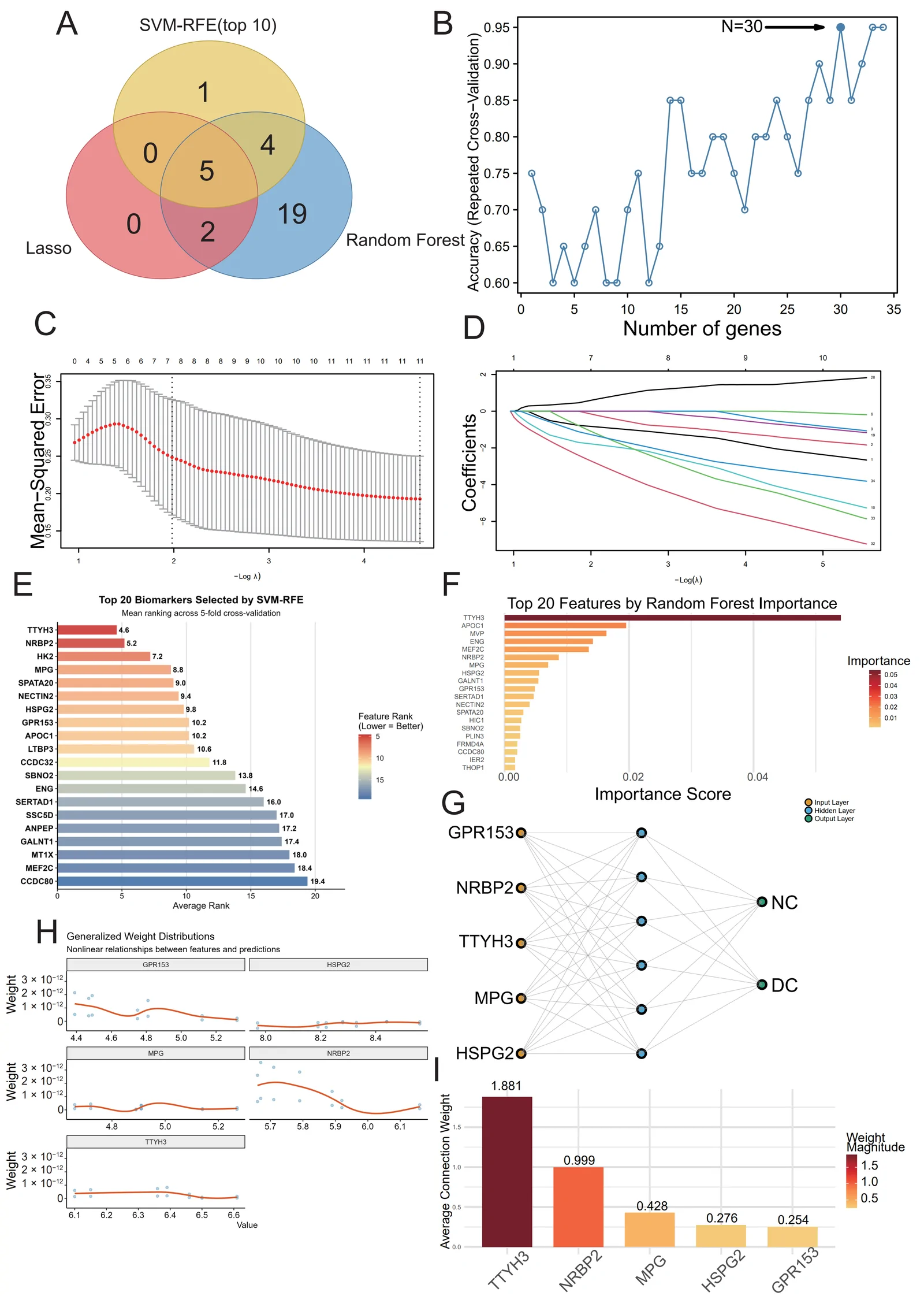
Machine learning methods for candidate genes: (**A**) The Venn diagram shows the selection of genes by machine learning methods. (**B**) Random forest prioritized 30 candidate genes. (**C**,**D**) The LASSO regression identified seven key genes. (**E**,**F**) The SVM-RFE and Random Forest algorithms, respectively rank the intersecting DEGs. (**G**) The visualization of ANN-based weighting analysis. (**H**) The weight of five key genes in ANN-based weighting analysis. (**I**) The Average Connection Weight ranking of five genes in the ANN-based weighting analysis.

To further prioritize the candidate osteoporosis-associated biomarkers of the intersecting DEGs, we applied three machine learning approaches—LASSO regression, Random Forest, and SVM-RFE—for exploratory feature selection, followed by an ANN-based weighting analysis (Figure 4A).

Based on the output results of LASSO regression, we identified seven genes with non-zero coefficients including one downregulated gene MEF2C and six upregulated genes TTYH3, NRBP2, MPG, ENG, GPR153, and HSPG2, with the penalty parameter selected by internal cross-validation (Figure 4C,D). The Random Forest algorithm was also applied, prioritizing 30 genes based on their feature-importance scores, with the number of retained features evaluated using repeated cross-validation (Figure 4B). SVM-RFE ranked the candidate genes according to their mean ranks across five-fold cross-validation and the top 20 genes were visualized (Figure 4E). The intersection of the top 10 genes identified by SVM-RFE, the top 30 genes ranked by the Random Forest algorithm (Figure 4F), and the seven genes selected by LASSO regression (Figure 4A) yielded five common candidate genes: TTYH3, NRBP2, MPG, HSPG2, and GPR153. An ANN-based weighting analysis was subsequently performed to characterize the relative contributions of these five preselected genes (Figure 4G). The generalized weight distributions were examined descriptively to visualize the nonlinear relationships between individual gene features and the ANN output (Figure 4H). The five genes were subsequently ranked according to their average connection weights derived from the ANN-based weighting analysis (Figure 4I).

Integration with the WGCNA results showed that TTYH3 and MPG were also present among the genes retained from the selected module. Therefore, we selected TTYH3 and MPG as the candidate osteoporosis-associated biomarkers in osteoblasts.

### 3.4. Subcluster Analysis, Pseudotime Trajectory, and CellChat Analysis of TTYH3 and MPG

**Figure 5:**
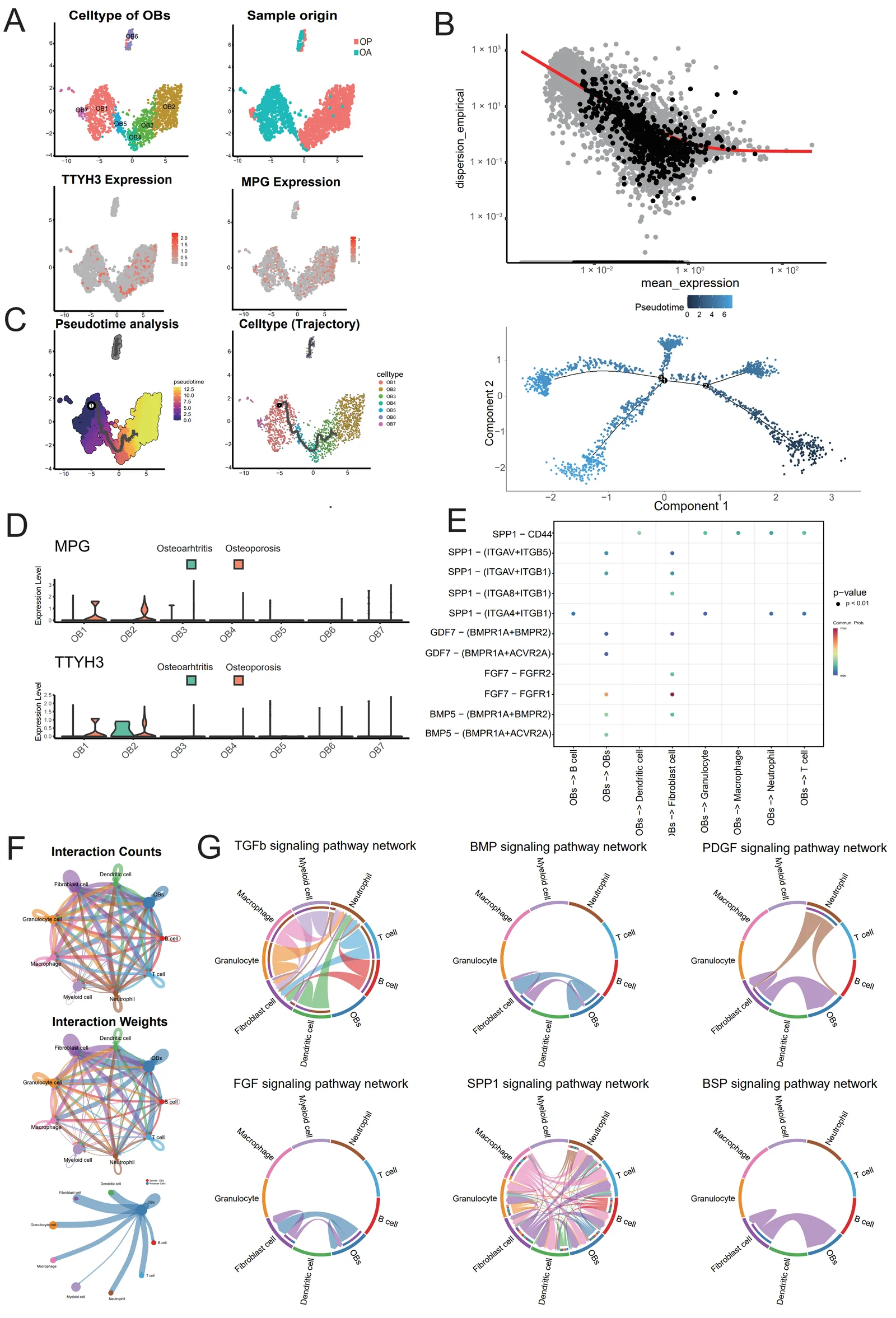
Subcluster analysis, pseudotime trajectory, and CellChat analysis: (**A**) Four UMAP plots illustrating subcluster distribution, group classification, and the expression patterns of TTYH3 and MPG across subclusters. (**B**) Pseudotime trajectory analysis of the OBs subcluster. The red line shows the fitted dispersion trend, and the dark and grey dots indicate selected and unselected genes, respectively. The numbers mark trajectory branch points. (**C**) UMAP plots depicting pseudotime differentiation trajectories and trajectory activity. (**D**) Violin plots showing the expression patterns of TTYH3 and MPG across subclusters. (**E**,**G**) Communication intensity of the six osteoporosis-associated ligand–receptor pathways among different cell types. (**F**) Circle plots displaying the strength and number of cell–cell interactions, with detailed communication patterns between OBs and other cell types.

Given the critical role of osteoblasts in regulating osteoclastogenesis and osteoporosis-related signaling pathways, we performed subcluster analysis of OBs. Using 15 principal components (PCs) and a resolution of 0.5, seven putative OB subclusters were identified and visualized using UMAP. OB1–OB7 contained 579, 571, 290, 109, 81, 75, and 69 cells, respectively. The expression patterns of TTYH3 and MPG were visualized on UMAP embeddings. OB2, OB3, and OB4 consisted predominantly of cells from the OP sample, whereas OB1 and OB5 were mainly derived from the OA sample; OB6 and OB7 contained cells from both samples (Figure 5A). The expression patterns of TTYH3 and MPG across the OB subclusters were further evaluated using violin plots (Figure 5D). Notably, OB2 exhibited markedly higher expression of TTYH3 and MPG. TTYH3 and MPG were detected in 26.6% and 40.1% of OB2 cells, respectively, compared with 10.4% and 16.3% of cells in the remaining subclusters. Moreover, 11.4% of OB2 cells co-expressed TTYH3 and MPG.

The inferred trajectory was consistent with the distribution patterns of the seven OB subclusters. Pseudotime trajectory analysis was performed using the Monocle 2 and Monocle 3 R packages to characterize the inferred transcriptional-state trajectory of individual cells (Figure 5B). The inferred trajectories were visualized using UMAP plots (Figure 5C). OB1 was identified as the root-associated state, whereas OB2 and OB3 were identified as distal trajectory state. The distinct trajectory position and transcriptional profile of OB2 suggest that this subcluster may represent a transcriptionally distinct state relative to the other OB subclusters.

To characterize cell–cell communication within femoral head cell populations, CellChat analysis was performed to reconstruct ligand–receptor interaction networks among different cell types. A total of 1533 cell-pair-specific ligand–receptor interaction records, representing 197 unique ligand–receptor combinations across 65 signaling pathways, were retained for exploratory network characterization. Circle plots were used to visualize the strength of within- and between-cluster communication across all cell types (Supplementary Figure S3C). Focusing on OBs, the number and strength of interactions between the OBs subcluster and other cell types were further examined and visualized (Figure 5F). The enrichment patterns of all 65 signaling pathways are presented in Supplementary Figure S4. Based on previously reported osteoporosis-related mechanisms, six candidate pathways potentially associated with OP were selected for further analysis: TGF-β, BMP, PDGF, FGF, SPP1, and BSP signaling pathways (Figure 5G). Ligand–receptor interactions between OBs and other cell types within these pathways are illustrated in Figure 5E.

### 3.5. Spatial Transcriptomic Profiling of Human Femoral Head

**Figure 6:**
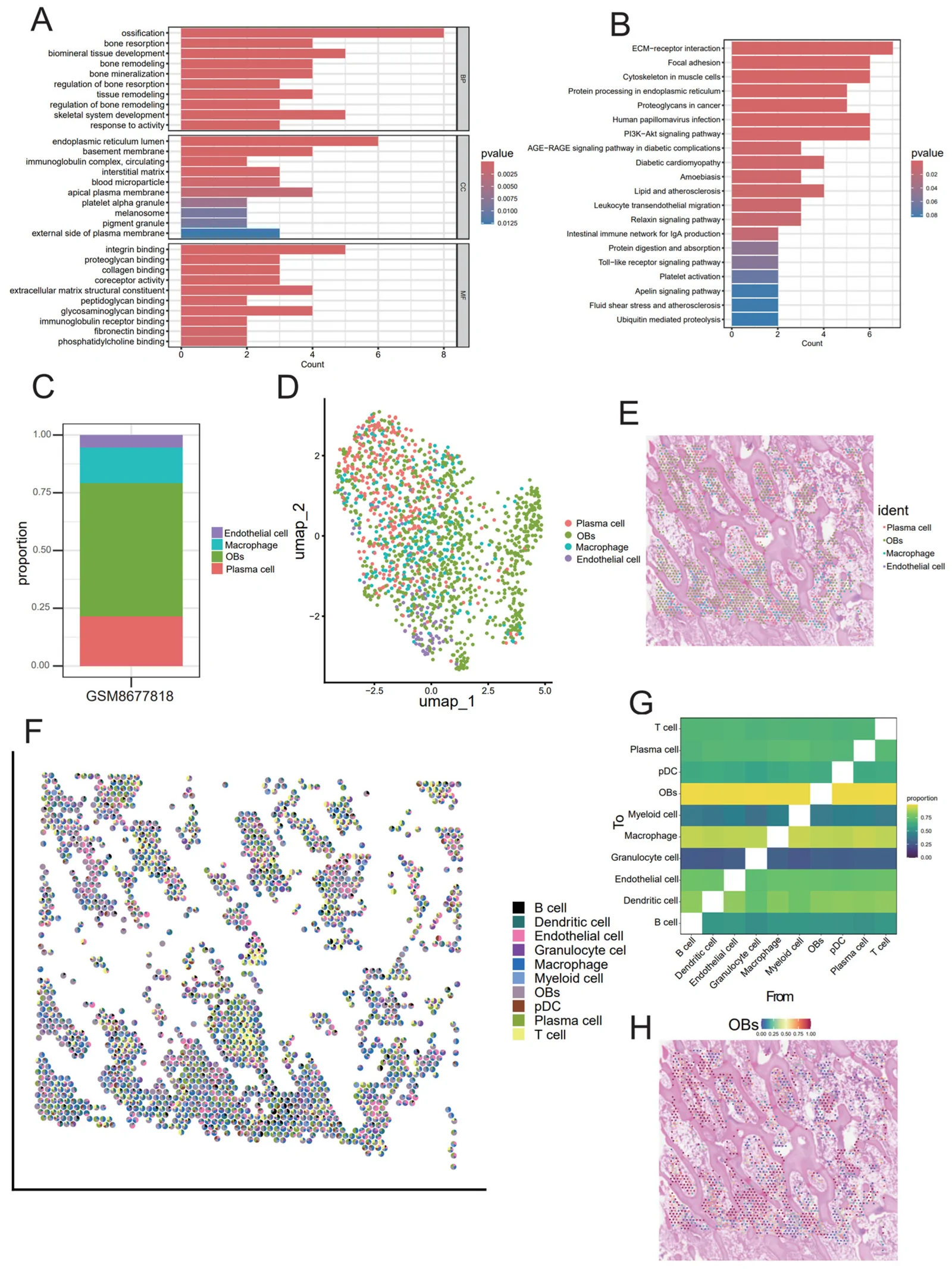
Spatial deconvolution analysis and characterization of cellular composition in spatial transcriptomics: (**A**,**B**) GO and KEGG enrichment analysis of cluster marker genes. (**C**) SPOTlight-based deconvolution using the GSE169396 scRNA-seq dataset as a reference to estimate cell proportions. (**D**,**E**) UMAP visualization and spatial distribution of deconvoluted cell populations. (**F**) Spatial scatter pie plots showing heterogeneous cellular composition across spots. (**G**) Spatial interaction analysis illustrating co-localization patterns among cell populations. (**H**) Spatial distribution of OBs highlighting their regional enrichment within the tissue section.

Raw spatial transcriptomic data from sample GSM8677818 were processed using Seurat. The initial dataset comprised expression measurements for 18,085 genes across 4992 spatial spots. After spots without detectable transcripts were excluded, 4849 spots underwent quality control based on the number of detected genes, UMI counts, and mitochondrial gene percentage (200 < nFeature_Spatial < 10,000, nCount_Spatial > 200, and mitochondrial gene percentage < 20%). A total of 1611 high-quality spots were retained for downstream analysis. Following SCTransform normalization, PCA was performed, and the first 40 principal components were used for UMAP visualization and Louvain clustering, which identified six spatial clusters. Cluster marker analysis using FindAllMarkers identified 2710 cluster marker genes, of which 598 were retained as significant cluster marker genes based on |avg_log2FC| > 1 and an adjusted *p* value < 0.05. GO enrichment analysis indicated that these genes were primarily associated with ossification, biomineral tissue development, and extracellular matrix organization, whereas KEGG enrichment analysis highlighted ECM–receptor interaction and focal adhesion pathways, indicating spatially distinct patterns of extracellular matrix remodeling and mineralization (Figure 6A,B). Six SVGs—COL1A1, IGHG3, CTSK, IGLC1, IGHD, and SPP1—were identified. Among them, SPP1 was also a top-ranked cluster marker gene and exhibited pronounced spatial heterogeneity.

To further characterize cellular composition and spatial distribution, SPOTlight deconvolution analysis was performed using the GSE169396 scRNA-seq dataset as a reference. NMF-based regression estimated the proportions of major cell populations, including osteoblasts (OBs), plasma cells, macrophages, and endothelial cells (Figure 6C). UMAP visualization confirmed distinct cellular profiles, whereas spatial mapping revealed heterogeneous localization patterns across the tissue section (Figure 6D,E). Spatial scatter pie plots further demonstrated the complex cellular composition of individual spots (Figure 6F), while spatial interaction analysis revealed potential co-localization patterns among different cell populations (Figure 6G). Notably, osteoblasts showed preferential spatial enrichment in mineralized tissue regions, highlighting their potential role in bone formation and remodeling (Figure 6H).

### 3.6. Spatial Identification of an SPP1-Enriched Osteogenic Region

**Figure 7:**
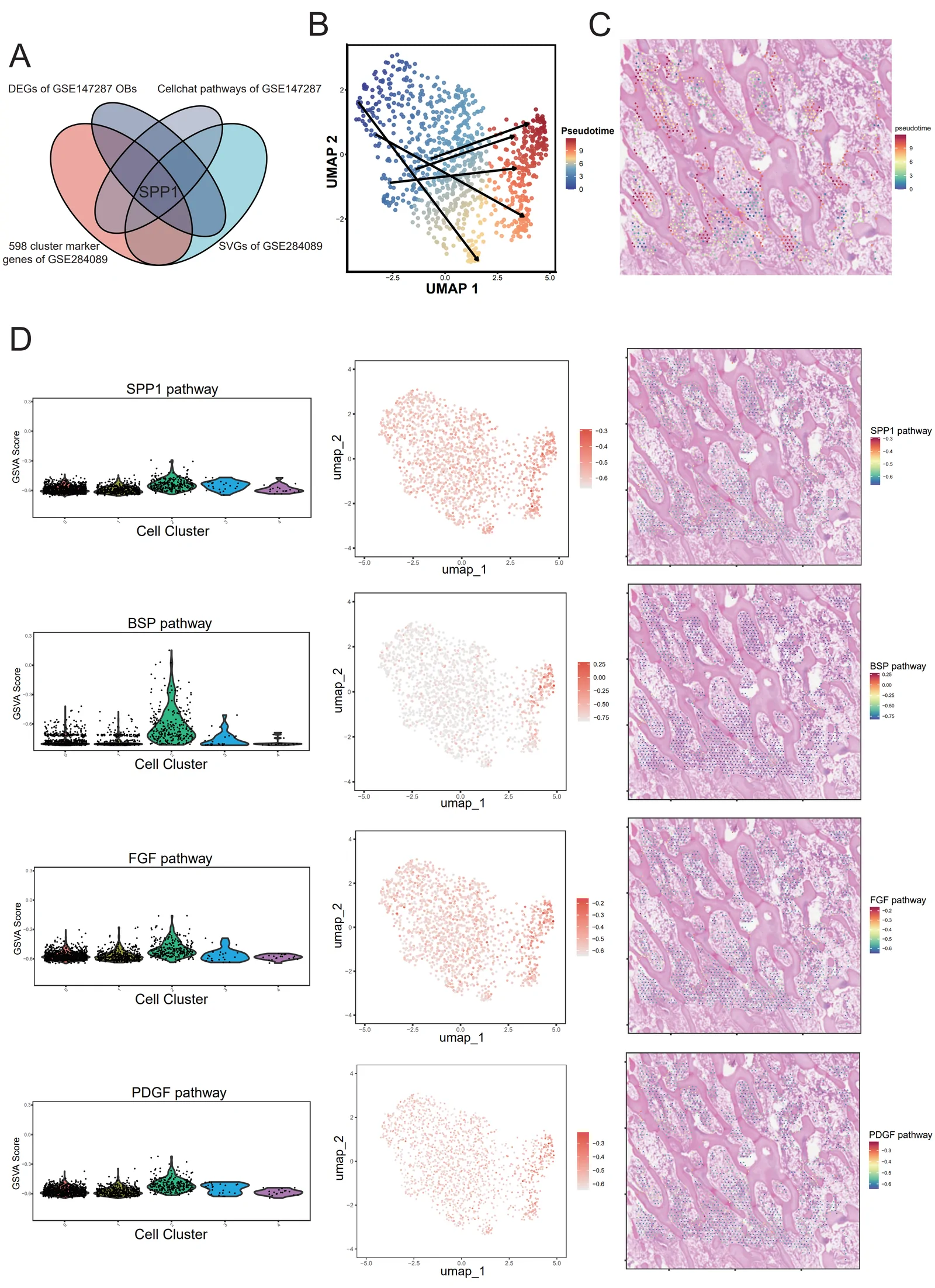
Identification of an osteoporosis-associated SPP1-enriched osteogenic region by spatial trajectory analysis: (**A**) Venn diagram showing the intersection of osteoporosis-related osteoblast DEGs from GSE147287, CellChat-derived signaling pathways, cluster marker genes, and spatially variable genes from GSE284089, identifying SPP1 as a convergent candidate gene. (**B**,**C**) Cell trajectory analysis showing transcriptional progression of spatial spots (**B**) and tissue coordinates (**C**), characterizing preferential enrichment of the SPP1-enriched region. The black arrows indicate the directions of pseudotime progression in the spatial trajectory analysis. (**D**) ssGSEA/GSVA of SPP1-, BSP-, FGF-, and PDGF-related pathways across spatial clusters.

To identify osteogenic programs with spatial and disease-related associations, we integrated osteoblast DEGs from GSE147287, CellChat-derived signaling pathways, cluster marker genes, and SVGs from GSE284089. The convergence of these four analytical components identified SPP1 as a candidate gene (Figure 7A). The signaling roles of individual cell types and the inferred intercellular communication network mediated by SPP1 are shown in Supplementary Figure S3A,B, respectively.

To further characterize the spatial transcriptional states associated with osteoporotic bone remodeling, cell trajectory analysis was performed based on spatial transcriptomic profiles. The reconstructed trajectory revealed a continuous transcriptional transition among spatial spots and identified a distinct terminal-like population separated from the major trajectory branch (Figure 7B). Importantly, spatial projection of pseudotime values demonstrated that this population was not randomly distributed throughout the tissue but exhibited a highly specific spatial pattern, with preferential enrichment along the outer surface of trabecular structures rather than within the trabecular core on hematoxylin and eosin (H&E)-stained tissue sections (Figure 7C). Combined with the spatial heterogeneity of SPP1 in GSE284089 and its higher expression in osteoporosis for osteoblasts in GSE147287, these findings suggest that this spatial domain may represent an osteoporosis-associated osteogenic state characterized by abnormal extracellular matrix remodeling and altered cell–matrix interactions.

Furthermore, four candidate signals derived from the osteoporosis-related cell communication pathways displayed coordinated enrichment within this terminal-like population. GSVA and ssGSEA analyses characterized enhanced activation of biological programs associated with these genes, while UMAP visualization and spatial mapping confirmed their preferential localization within the SPP1-enriched region (Figure 7D). Together, these results may characterize a spatially restricted SPP1-enriched osteogenic region associated with osteoporosis progression and highlight a potential regulatory module linking intercellular communication, spatial transcriptional heterogeneity, and pathological bone remodeling.

### 3.7. Immunohistochemical Staining by Clinical Samples and Experimental Validation

**Figure 8:**
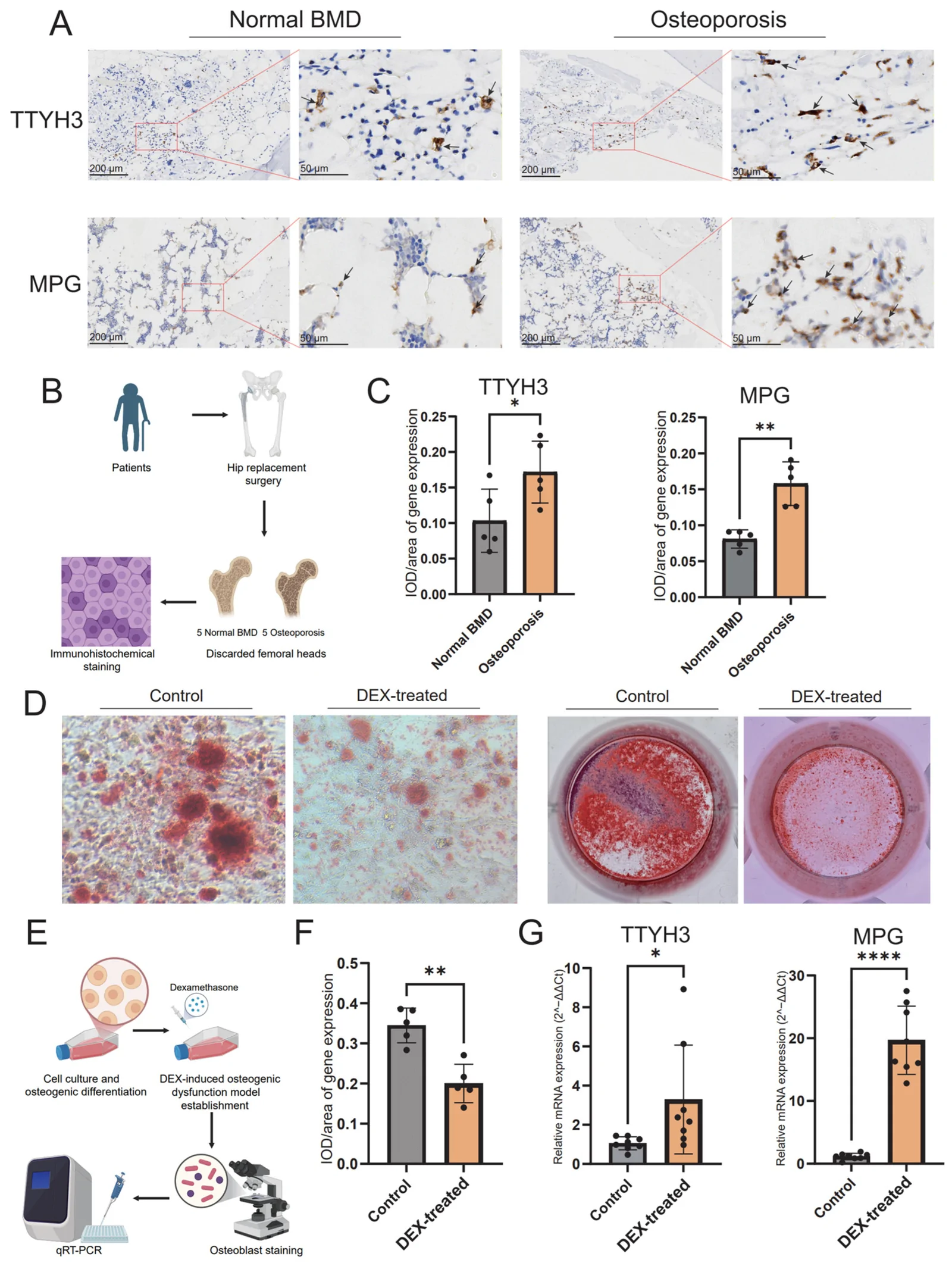
Immunohistochemical staining by clinical samples and experimental validation: (**A**) Representative immunohistochemical staining images of TTYH3 and MPG in samples. (**B**) Experimental procedures of immunohistochemical staining. (**C**) Quantitative statistical analysis of TTYH3 and MPG. (**D**) 1 μM DEX induced the DEX-induced osteogenic dysfunction model of MC3T3-E1 cells and Alizarin Red S staining. (**E**) Experimental procedures of DEX-induced osteogenic dysfunction model construction and qRT-PCR. (**F**) Quantitative statistical analysis of Alizarin Red S staining between the control group and DEX-treated group. (**G**) TTYH3 and MPG were highly expressed in the DEX-treated group by qRT-PCR. (* represents *p* value < 0.05, ** represents *p* value < 0.01, *** represents *p* value < 0.001, **** represents *p* value < 0.0001; IHC, n = 5 patients per group; qRT-PCR, n = 8 per group).

To validate the expression of TTYH3 and MPG in human bone tissues, discarded femoral head specimens were collected from patients undergoing total hip arthroplasty and analyzed by immunohistochemical staining. The immunohistochemical staining workflow is shown in Figure 8B. Compared with femoral head tissues from individuals with normal bone mass, osteoporotic samples exhibited markedly enhanced staining intensity for both TTYH3 and MPG (Figure 8A). Quantitative analysis further demonstrated that the protein expression levels of TTYH3 and MPG were significantly increased in osteoporotic femoral head tissues, with MPG showing a more pronounced elevation than TTYH3 (Figure 8C). These findings indicate that TTYH3 and MPG are upregulated in human osteoporotic bone tissues, suggesting their potential involvement in osteoporosis-associated bone alterations.

To further investigate the potential role of TTYH3 and MPG during osteoporosis progression, an in vitro model of DEX-induced osteogenic dysfunction was established using MC3T3-E1 cells treated with DEX. Following osteogenic induction, matrix mineralization was evaluated by Alizarin Red S staining. The experimental workflow is shown in Figure 8E. Compared with control cells, DEX-treated cells exhibited a marked reduction in mineralized nodule formation, as evidenced by weaker Alizarin Red S staining intensity and fewer mineralized deposits (Figure 8D). Quantitative analysis of Alizarin Red S staining from five randomly selected microscopic fields further confirmed that mineralization capacity was significantly decreased in the DEX-treated group compared with the control group (Figure 8F), indicating impaired osteogenic differentiation following DEX exposure.

To determine whether the expression patterns of TTYH3 and MPG were altered under conditions of DEX-induced osteogenic dysfunction, qRT-PCR analysis was performed. Compared with the control group, DEX-treated osteoblasts displayed significantly elevated mRNA levels of TTYH3 and MPG (Figure 8G). In particular, MPG expression showed a pronounced increase, whereas TTYH3 expression was also significantly upregulated after DEX exposure. These results demonstrate that the DEX treatment suppressed osteogenic mineralization while inducing the expression of TTYH3 and MPG, suggesting that both genes may be involved in the regulation of osteoblast dysfunction during osteoporosis development.

## 4. Discussion

Osteoblasts are bone-forming cells essential for skeletal development, homeostasis, and repair. They synthesize and secrete type I collagen-rich osteoid, initiate its mineralization, regulate bone remodeling through paracrine signaling (e.g., RANKL/OPG), and ultimately undergo terminal differentiation into osteocytes or bone-lining cells. Osteoblast dysfunction is a central pathogenic driver of osteoporosis, characterized by reduced bone formation, impaired matrix quality, and disrupted coupling with osteoclast activity. Supporting this, Wang et al. demonstrated that osteoblast-specific Men1 deletion induces cortical bone loss in mice by triggering cellular senescence—evidenced by increased p16INK4a expression, diminished osteoblast numbers and surface coverage, and suppressed bone formation indices [31]. Similarly, Liu et al. showed that osteoblast-intrinsic vitamin D receptor (VDR) deficiency compromises collagen synthesis, delays mineralization kinetics, and leads to reduced bone mass and mechanical strength [32]. Furthermore, Jiang et al. established that the transcription factor Sp7 (Osterix) is indispensable for osteoblast proliferation, COL1A1 transcription, and cytoskeletal reorganization during osteocyte embedding; Sp7 ablation results in defective osteoblast function, disorganized bone architecture, and progressive trabecular and cortical bone loss [33].

In this study, we integrated scRNA-seq, bulk RNA-seq, and spatial transcriptomics (ST) data with bioinformatics methods, including differential expression analysis, machine learning, and WGCNA, to identify TTYH3 and MPG as candidate osteoporosis-associated biomarkers in osteoblasts and to characterize the spatial heterogeneity of SPP1 in the osteoporotic bone microenvironment. Subcluster, pseudotime trajectory, and CellChat analyses suggested their potential roles in osteoporosis pathogenesis. Immunohistochemical staining analysis of clinical samples confirmed elevated TTYH3 and MPG expression in human femoral head tissues from individuals with osteoporosis compared with those from individuals with normal bone mass. The qRT-PCR analysis further demonstrated their upregulation in a DEX-induced MC3T3-E1 cell model of impaired osteogenic function. Members of the Tweety family—particularly TTYH3—encode calcium-activated chloride channels, with TTYH3 exhibiting high expression in excitable tissues including neurons and skeletal muscle [34,35]. Ion channel-mediated calcium and chloride fluxes are critical regulators of osteoblast function and bone homeostasis; dysregulation contributes directly to osteoporotic pathogenesis. Specifically, L-type voltage-gated calcium channel (LTCC)-mediated Ca^2+^ influx activates calcineurin (CaN), triggering nuclear translocation of nuclear factor of activated T cells (NFAT) via dephosphorylation. Nuclear NFAT cooperates with the master osteogenic transcription factor Sp7 (Osterix) to drive transcription of key bone matrix genes—including osteocalcin (BGLAP) and collagen type I alpha 1 chain (COL1A1) [36]. Sun et al. showed that Ano1 (Anoctamin 1), a calcium-activated chloride channel highly expressed in osteoclast precursors, is essential for osteoclast differentiation, acid secretion, and bone resorption. Inhibiting Ano1 genetically or pharmacologically reduces osteoporosis in mice, validating it as a mechanistically supported therapeutic target. [37].

The role of TTYH3 in osteoporosis pathogenesis remains uncharacterized. In contrast, TTYH3 dysregulation has been implicated in multiple cancers—including cervical, hepatocellular, bladder, and non-small cell lung carcinomas—suggesting its broader involvement in cellular proliferation and survival pathways [38,39,40]. Mechanosensitive ion channels—including TREK-1, Piezo1/2, and volume-regulated anion channels (VRACs)—are emerging therapeutic targets for osteoporosis. By transducing mechanical cues into intracellular Ca^2+^ signals, they regulate osteoblast–osteoclast coupling and bone remodeling homeostasis [41,42]. Consistent with this, intracellular Ca^2+^ dynamics constitute a central regulatory hub for osteoblast function: Ca^2+^ influx through L-type voltage-gated calcium channels (LTCCs), Orai1/STIM-dependent store-operated calcium entry (SOCE), and TRP family channels orchestrates spatiotemporal signaling. Downstream effectors—including calmodulin, CaMKII, and the calcineurin–NFAT axis—integrate these signals to activate RUNX2 and Sp7 (Osterix), thereby driving osteogenic gene expression and matrix mineralization. Disruption of Ca^2+^ channel activity or Ca^2+^ homeostasis impairs osteoblast differentiation and bone formation, directly contributing to osteoporotic bone loss [43,44].

Building on prior evidence, we propose that TTYH3 overexpression may contribute to osteoblast dysfunction and osteoporosis progression by perturbing membrane electrophysiology and mechanotransduction. As a calcium-activated chloride channel, elevated TTYH3 enhances Cl^−^ efflux, disrupts cellular ion homeostasis, and destabilizes resting membrane potential. These alterations impair activation of mechanosensitive Ca^2+^ channels—including Piezo1 and TRPV4—thereby compromising Ca^2+^ signaling required for osteogenic differentiation. Consequently, downstream effectors such as the Wnt/β-catenin pathway, CaMKII, ERK, and RUNX2-mediated transcription are suppressed, leading to defective osteoblast differentiation and matrix mineralization [45]. Furthermore, excessive Cl^−^ efflux drives osmotic water loss and cell shrinkage, reducing membrane tension and disrupting cytoskeletal architecture—key determinants of mechanosensor sensitivity. This impairs osteoblast responsiveness to mechanical stimuli and attenuates mechanically evoked Ca^2+^ transients. Collectively, TTYH3 overexpression establishes a previously unrecognized nexus among chloride homeostasis, membrane biophysics, and Ca^2+^-dependent mechanotransduction in osteoblasts during osteoporosis pathogenesis [46,47].

The MPG-encoded N-methylpurine DNA glycosylase is a key DNA repair enzyme that maintains genomic stability, supports normal development, and contributes to disease pathogenesis [48]. The MPG gene primarily functions in the base excision repair (BER) pathway by recognizing and excising methylated DNA bases, such as 3-methyladenine and 7-methylguanine, representing the initial step of BER [49]. Osteoblast aging can lead to an imbalance in bone remodeling and promote bone loss. Osteoblast aging disrupts bone remodeling homeostasis and contributes to bone loss. Accumulated DNA damage is a key driver of osteoblast senescence, with studies indicating that DNA damage may accelerate bone aging through NF-κB signaling [50]. Consistent with this, DNA repair capacity declines with osteoblast aging, and MPG—alongside other BER components—plays a critical role in counteracting DNA damage accumulation to preserve osteogenic potential. Supporting the centrality of DNA repair in bone health, Dong et al. showed that SIRT6, an NAD^+^-dependent deacetylase, regulates osteoblast differentiation and survival by coordinating DNA repair, metabolic adaptation, and transcriptional activation of RUNX2 and Sp7 (Osterix); SIRT6 deficiency impairs osteoblast proliferation, enhances apoptosis, and exacerbates osteoporosis in vivo [51].

SPP1 encodes osteopontin (OPN), a multifunctional extracellular matrix protein critical for bone remodeling and osteoporosis pathogenesis. By binding integrins and CD44, SPP1 directly regulates osteoclast adhesion, migration, and bone resorption—thereby maintaining skeletal homeostasis [52]. As a central mediator of the bone microenvironment, SPP1 orchestrates intercellular crosstalk among osteoblasts, osteocytes, osteoclasts, and immune cells [53]. Recent scRNA-seq studies have uncovered profound cellular heterogeneity in osteoporotic bone, while spatial transcriptomics has revealed compartment-specific SPP1 expression—enriched in endosteal surfaces, bone marrow niches, and sites of active remodeling [54]. In this study, integrated analysis of scRNA-seq data (GSE147287) and ST data (GSE284089) further elucidated the role of SPP1 in osteoporosis. Beyond being a highly expressed osteoporosis-associated gene, SPP1 was identified as a key regulator of cell–cell communication networks with distinct spatial distribution patterns in bone tissues. These findings indicate that SPP1 may serve not only as a biomarker of osteoporosis but also as a spatially regulated signaling hub coordinating cellular interactions within the osteoporotic bone microenvironment.

Based on our multi-omics findings, TTYH3 and SPP1 were selected as key molecules for further mechanistic exploration. TTYH3 represented the highest-ranked candidate gene identified through the integration of single-cell RNA sequencing, bulk RNA-seq and machine learning approaches (Figure 4E–I), whereas SPP1 was identified among the top-ranked cluster marker genes in the combined single-cell and ST analyses (Figure 7A), providing a rationale for investigating their potential functional relationship in osteoporosis progression. Although TTYH3 and SPP1 have been individually implicated in cellular ion regulation and bone remodeling, respectively, their potential functional connection in osteoporosis remains unexplored. Based on our integrated transcriptomic and spatial analyses, we cautiously propose a hypothetical TTYH3–calcium signaling–SPP1 axis. In this model, TTYH3 may act as an upstream electrophysiological regulator associated with osteoblast dysfunction, whereas SPP1 may function as a downstream, spatially regulated effector within the osteoporotic bone microenvironment. Elevated TTYH3 expression could potentially disrupt chloride homeostasis and membrane electrophysiological properties, thereby influencing the activation of mechanosensitive calcium channels and calcium-dependent osteogenic signaling. Ion channels and mechanotransduction pathways, particularly Piezo1- and TRPV4-mediated calcium signaling, have been shown to play essential roles in osteoblast differentiation, bone formation, and skeletal adaptation to mechanical stimuli [41].

Given the critical role of intracellular calcium dynamics in regulating osteogenic transcriptional programs, persistent calcium signaling dysregulation could affect RUNX2- and Osterix-dependent gene expression and extracellular matrix regulation, potentially contributing to altered SPP1 expression. Previous studies have demonstrated that calcium-dependent pathways, including CaMKII, calcineurin–NFAT, and Wnt/β-catenin signaling, are central regulators of osteoblast differentiation and mineralization [36]. Accordingly, we speculate that TTYH3 upregulation may contribute to an unfavorable electrophysiological state associated with osteogenic dysfunction and SPP1-related remodeling responses during osteoporosis progression. However, the directionality and causal relationships within this proposed axis remain unverified and require further mechanistic investigation.

Importantly, the spatial characteristics of SPP1 identified in our study provide further insights into the regional vulnerability of osteoporotic bone. Bone remodeling is highly heterogeneous, with mechanically active regions and remodeling niches exhibiting distinct cellular compositions and molecular signatures. Recent scRNA-seq and spatial transcriptomic studies have demonstrated extensive cellular heterogeneity and spatial organization within skeletal tissues, emphasizing the importance of spatial regulation in understanding bone homeostasis and disease progression [55,56].

The spatial enrichment of SPP1 in osteoporotic bone may reflect a localized response to altered mechanical stimulation, impaired calcium signaling and disrupted osteoblast–osteoclast communication. SPP1 is known to regulate osteoclast adhesion, migration, and bone remodeling through integrin and CD44-dependent signaling, and its dysregulation may contribute to abnormal osteoimmune interactions within the bone microenvironment [57]. Increased SPP1 expression may therefore amplify extracellular matrix remodeling and reinforce the imbalance between bone formation and resorption. Collectively, our findings support the hypothesis of a potential TTYH3–calcium signaling–SPP1 regulatory axis. In this proposed model, altered membrane electrophysiology and spatially coordinated extracellular matrix responses may converge to impair osteoblast function and contribute to osteoporosis progression. Although this hypothesis requires further experimental validation, it provides a conceptual framework linking ion channel-associated regulation, impaired mechanotransduction, and spatial remodeling abnormalities, supporting further investigation of TTYH3 and SPP1 as candidate biomarkers and potential therapeutic targets for osteoporosis.

An important limitation of this study is the use of OA-derived specimens as comparators in the scRNA-seq and bulk RNA-seq analyses. OA cannot be regarded as a genuinely healthy condition because OA-associated subchondral sclerosis, inflammation, altered mechanical loading, and changes in local bone turnover may independently affect osteoblast gene expression [58]. Accordingly, the observed transcriptional differences should be interpreted as differences between the OP and OA specimens rather than as definitive osteoporosis-specific alterations relative to healthy bone [59].

Nevertheless, the selection of OA-derived femoral head tissue has practical justification given the clinical and ethical constraints associated with obtaining human bone specimens. Healthy individuals with normal bone mass have no clinical indication for femoral head removal, making genuinely healthy femoral head tissue extremely difficult to obtain. Other surgically available tissues, including those from patients with osteonecrosis, femoral neck fracture, developmental dysplasia, or ankylosing spondylitis, also involve substantial pathological confounding [60,61]. Therefore, OA-derived femoral head tissue with clinically confirmed normal bone mass may serve as a pragmatic disease comparator, although it should not be described as a healthy control. Future studies using well-characterized non-OA comparators will be required to determine whether the identified molecular alterations are specific to osteoporosis.

## 5. Conclusions

In conclusion, we integrated scRNA-seq, bulk RNA-seq, and ST to systematically characterize molecular and cellular alterations associated with osteoporosis. Differential expression analysis, WGCNA, and machine learning approaches identified TTYH3 and MPG as candidate genes associated with osteoporosis. Spatial transcriptomic analysis further characterized the spatial distribution of SPP1 and highlighted its potential involvement in the osteoporotic bone microenvironment. The expression patterns of TTYH3 and MPG were further validated by immunohistochemical staining of human femoral head samples and qRT-PCR analysis of MC3T3-E1 cells in a DEX-induced osteogenic dysfunction model. Collectively, our study provides a comprehensive multi-omics framework for investigating osteoporosis-associated molecular alterations and proposes a potential TTYH3–calcium signaling–SPP1 regulatory axis that may contribute to osteogenic dysfunction and disease progression. These findings identify TTYH3 and MPG as candidate osteoporosis-associated biomarkers and SPP1 as a spatially heterogeneous molecule of interest, warranting further mechanistic and clinical validation.

## Data Availability

The data analyzed in this study were derived from publicly accessible repositories. They can be download from Gene Expression Omnibus (https://www.ncbi.nlm.nih.gov/geo/, accessed on 18 August 2026) at series number GSE147287, GSE156508, GSE284089, and GSE169396.

## Supplementary Materials

The following supporting information can be downloaded at: https://www.mdpi.com/article/10.3390/biomedicines14092126/s1, Figure S1: The processing procedure of scRNA-seq data. Figure S2: Clustering of scRNA-seq data and cellmarkers for clusters. Figure S3: Cellchat analysis and visualization related to the SPP1 pathway. Figure S4: Cellchat analysis reveals all the ligand-receptor pathways that have been enriched.

## Abbreviations

The following abbreviations are used in this manuscript:

α-MEM	Alpha Minimum Essential Medium
ALPL	Alkaline phosphatase, liver/bone/kidney
ANN	Artificial neural network
BER	Base excision repair
BMD	Bone mineral density
BMP	Bone morphogenetic protein
BSP	Bone sialoprotein
CaMKII	Ca^2+^/calmodulin-dependent protein kinase II
CaN	Calcineurin
COL1A1	Collagen type I alpha 1 chain
DAB	3,3′-Diaminobenzidine
DEGs	Differentially expressed genes
DEX	Dexamethasone
ECM	Extracellular matrix
EDTA	Ethylenediaminetetraacetic acid
ENG	Endoglin
ERK	Extracellular signal-regulated kinase
FBS	Fetal bovine serum
FC/logFC	Fold change/log fold change
FGF	Fibroblast growth factor
GEO	Gene Expression Omnibus
GMT	Gene Matrix Transposed
GO	Gene Ontology
GPR153	G protein-coupled receptor 153
GSVA	Gene Set Variation Analysis
HDF5	Hierarchical Data Format version 5
HSC	Hematopoietic stem cell
HSPG2	Heparan sulfate proteoglycan 2
H&E	Hematoxylin and eosin
IEC	Institutional Ethics Committee
IHC	Immunohistochemical staining
IOD	Integrated optical density
KEGG	Kyoto Encyclopedia of Genes and Genomes
LASSO	Least Absolute Shrinkage and Selection Operator
LTCC	L-type voltage-gated calcium channel
MEF2C	Myocyte enhancer factor 2C
MEs	Module eigengenes
MPG	N-methylpurine DNA glycosylase
MSC	Mesenchymal stromal cell
NAD^+^	Nicotinamide adenine dinucleotide
NFAT	Nuclear factor-activated T cells
NF-κB	Nuclear factor kappa-light-chain-enhancer of activated B cells
NGFR	Nerve growth factor receptor
NMF	Non-negative matrix factorization
NRBP2	Nuclear receptor binding protein 2
OA	Osteoarthritis
OBs	Osteoblasts
OCN	Osteocalcin
OIM	Osteogenic induction medium
OP	Osteoporosis
OPN	Osteopontin
OPG	Osteoprotegerin
PCA	Principal component analysis
PCs	Principal components
PBS	Phosphate-buffered saline
PDGF	Platelet-derived growth factor
Piezo1	Piezo type mechanosensitive ion channel component 1
PMOP	Postmenopausal osteoporosis
PPI	Protein–Protein Interaction
qCT	Quantitative computed tomography
qRT-PCR	Quantitative real-time polymerase chain reaction
RANKL	Receptor activator of nuclear factor kappa-B ligand
RUNX2	Runt-related transcription factor 2
SCT	SCTransform
SD	Standard deviation
SIRT6	Sirtuin 6
SPARC	Secreted protein acidic and cysteine rich
SPP1	Secreted phosphoprotein 1 (osteopontin)
Sp7	Sp7 transcription factor
ssGSEA	Single-sample Gene Set Enrichment Analysis
ST	Spatial transcriptomics
SVM-RFE	Support Vector Machine Recursive Feature Elimination
SVGs	Spatially variable genes
TGF-β	Transforming growth factor beta
TOM	Topological overlap matrix
TRP	Transient receptor potential
TRPV4	Transient receptor potential vanilloid 4
t-SNE	T-Distributed Stochastic Neighbor Embedding
TTYH3	Tweety family member 3
UMI	Unique molecular identifier
UMAP	Uniform Manifold Approximation and Projection
VDR	Vitamin D receptor
VRACs	Volume-regulated anion channels
WGCNA	Weighted gene co-expression network analysis
