# Supplementary material for: Multi-Omics Analysis Identifies TTYH3 and MPG as Candidate Osteoporosis-Associated Biomarkers in Osteoblasts and Characterizes Spatial Heterogeneity of SPP1 in the Osteoporotic Bone Microenvironment
Source: Biomedicines. 2026 Sep 20;14(9):2126. doi: 10.3390/biomedicines14092126 (PMC13604504; doi:10.3390/biomedicines14092126)
Supplement: Supplementary file 1 [file biomedicines-14-02126-s001.zip › biomedicines-4542916-supplementary.pdf]

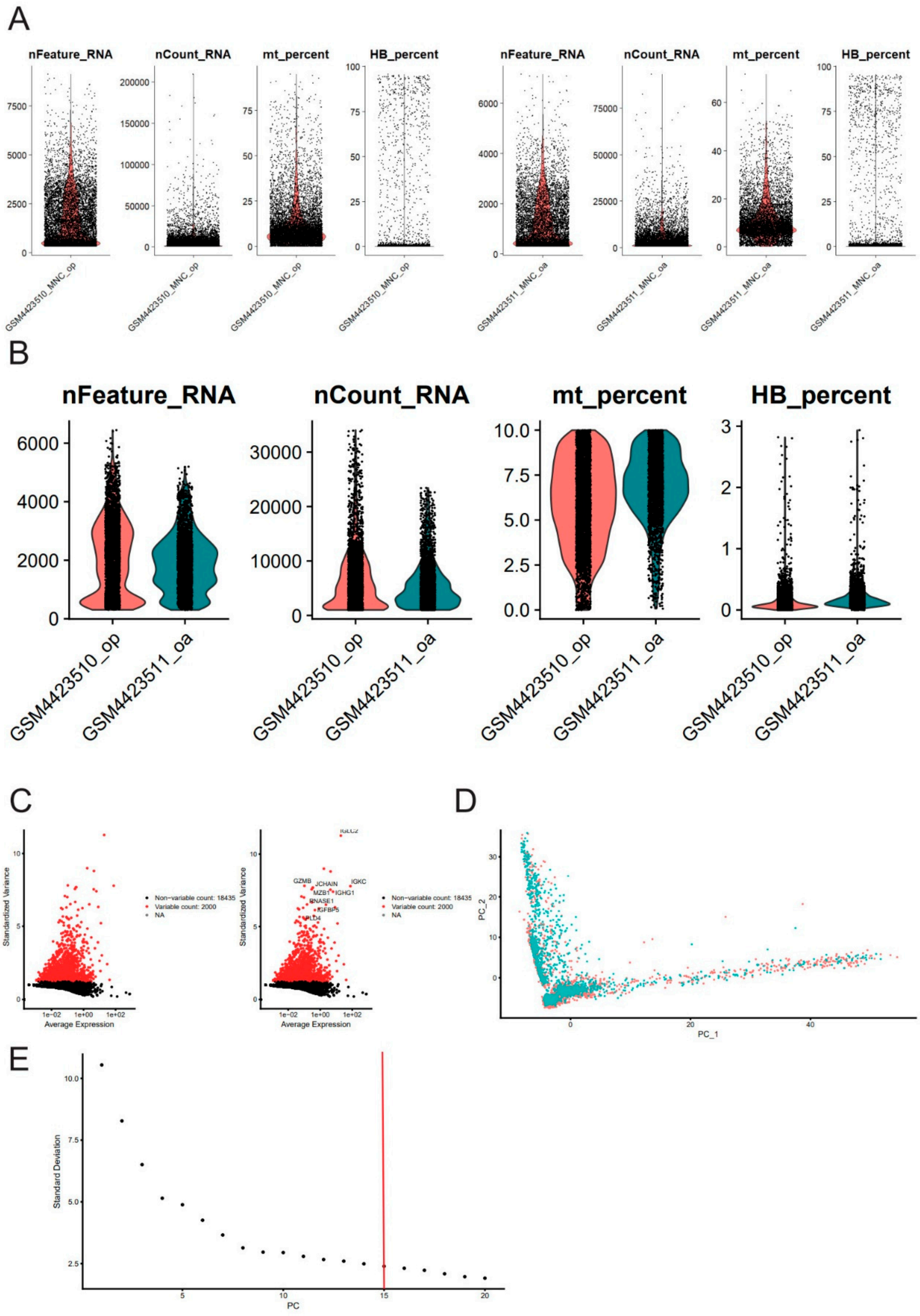

Supplementary Figure S1. The processing procedure of scRNA-seq data: (**A**) The feature of RNA, count of RNA, percentage of mitochondrial genes, and percentage of HB genes before quality control. (**B**) The violin plots show the four indicators after quality control. (**C,D**) Search for highly variable genes. (**E**) Principal component analysis (PCA) and the search of significant principal component.

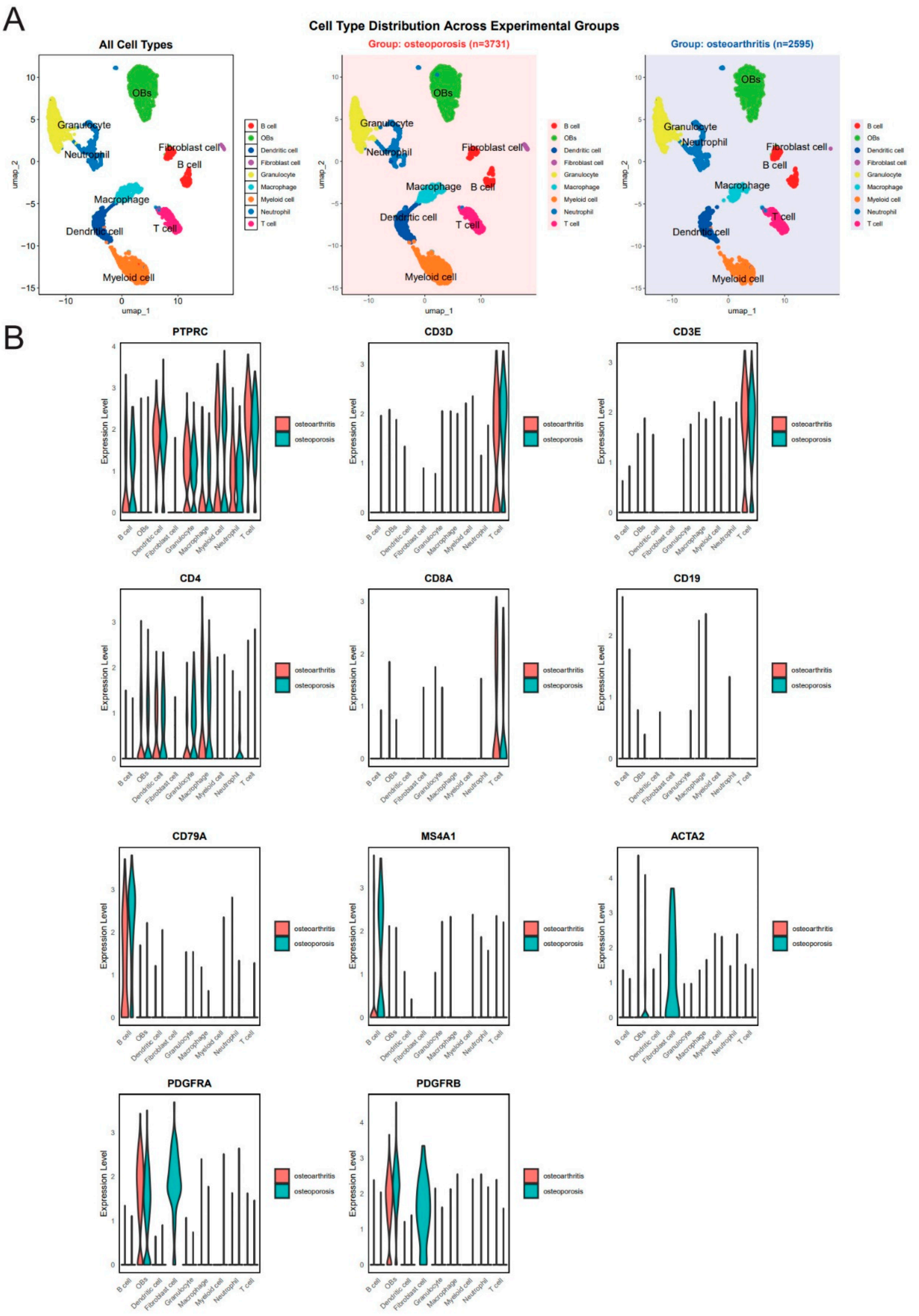

Supplementary Figure S2. Clustering of scRNA-seq data and cellmarkers for clusters: (**A**) Different clusters are visualized using UMAP diagrams and each shows osteoporosis, osteoarthritis, and the combined diagram of the two samples. (**B**) Typical cellmarkers of different clusters are shown in violin plots.

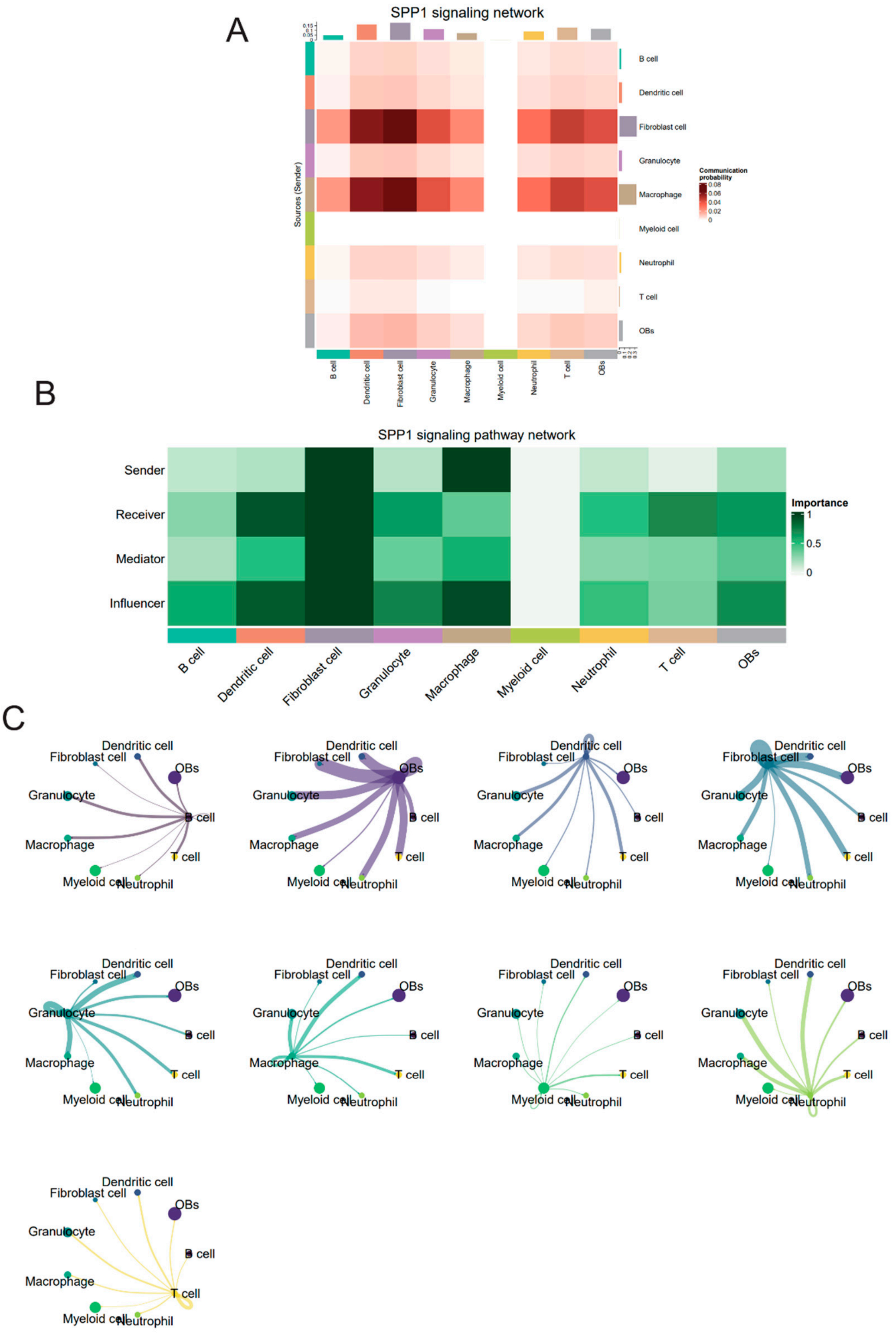

Supplementary Figure S3. Cellchat analysis and visualization related to the SPP1 pathway: (**A**,**B**) The distribution of SPP1 pathway ligands and receptors in different clusters. (**C**) Different clusters were respectively visualized using circle plots to show their intercellular cellchat situations.

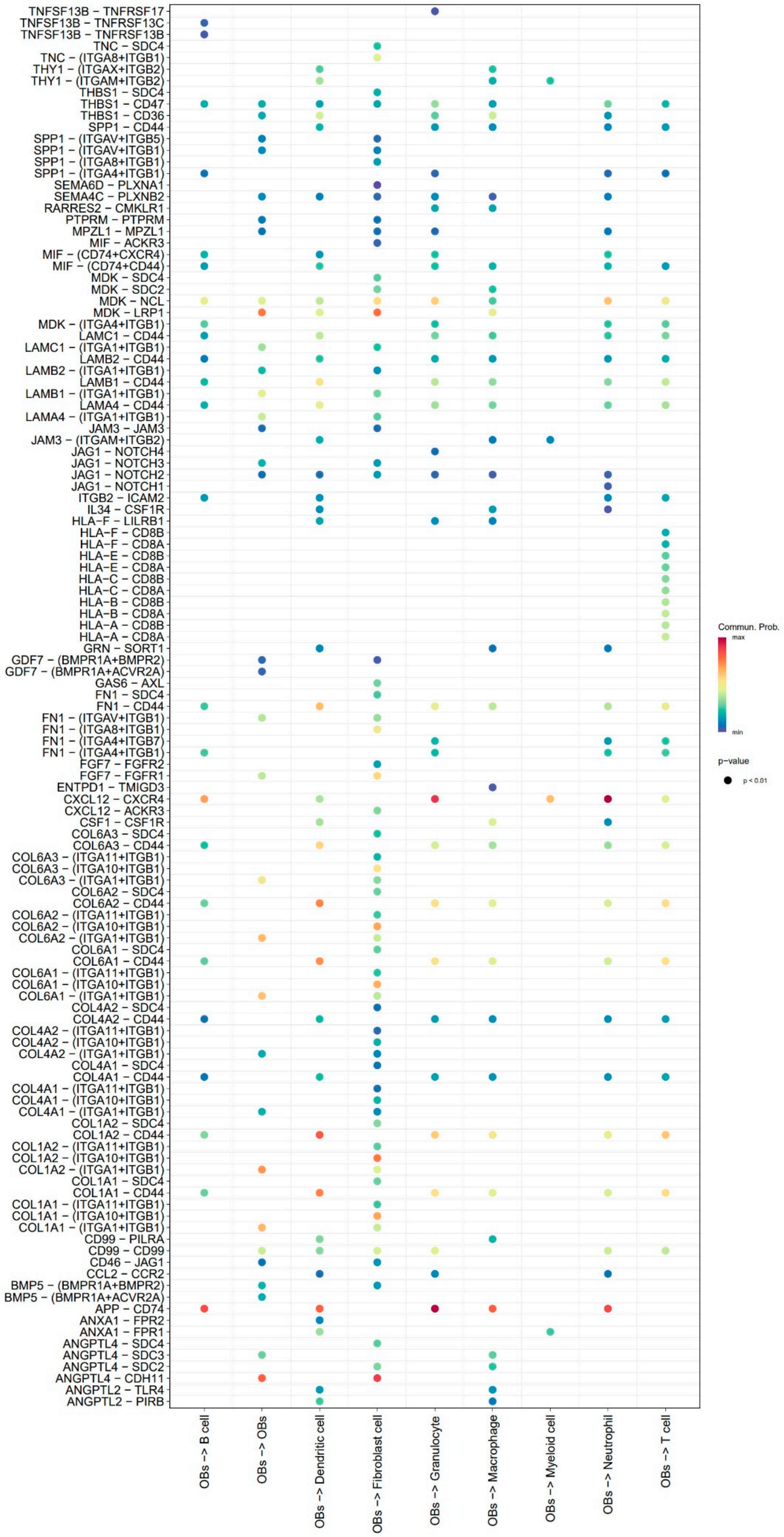
TNFSF13B – TNFRSF17
TNFSF13B – TNFRSF13C
TNFSF13B – TNFRSF13B
TNC – SDC4
TNC – (ITGA8+ITGB1)
THY1 – (ITGAX+ITGB2)
THY1 – (ITGAM+ITGB2)
THBS1 – SDC4
THBS1 – CD47
THBS1 – CD36
SPP1 – CD44
SPP1 – (ITGAV+ITGB5)
SPP1 – (ITGAV+ITGB1)
SPP1 – (ITGA8+ITGB1)
SPP1 – (ITGA4+ITGB1)
SEMA6D – PLXNA1
SEMA4C – PLXNB2
RARRES2 – CMKLR1
PTPRM – PTPRM
MPZL1 – MPZL1
MIF – ACKR3
MIF – (CD74+CXCR4)
MIF – (CD74+CD44)
MDK – SDC4
MDK – SDC2
MDK – NCL
MDK – LRP1
MDK – (ITGA4+ITGB1)
LAMC1 – CD44
LAMC1 – (ITGA1+ITGB1)
LAMB2 – CD44
LAMB2 – (ITGA1+ITGB1)
LAMB1 – CD44
LAMB1 – (ITGA1+ITGB1)
LAMA4 – CD44
LAMA4 – (ITGA1+ITGB1)
JAM3 – JAM3
JAM3 – (ITGAM+ITGB2)
JAG1 – NOTCH4
JAG1 – NOTCH3
JAG1 – NOTCH2
JAG1 – NOTCH1
ITGB2 – ICAM2
IL34 – CSF1R
HLA–F – LILRB1
HLA–F – CD8B
HLA–F – CD8A
HLA–E – CD8B
HLA–E – CD8A
HLA–C – CD8B
HLA–C – CD8A
HLA–B – CD8B
HLA–B – CD8A
HLA–A – CD8B
HLA–A – CD8A
GRN – SORT1
GDF7 – (BMPR1A+BMPR2)
GDF7 – (BMPR1A+ACVR2A)
GAS6 – AXL
FN1 – SDC4
FN1 – CD44
FN1 – (ITGAV+ITGB1)
FN1 – (ITGA8+ITGB1)
FN1 – (ITGA4+ITGB7)
FN1 – (ITGA4+ITGB1)
FGF7 – FGFR2
FGF7 – FGFR1
ENTPD1 – TMIGD3
CXCL12 – CXCR4
CXCL12 – ACKR3
CSF1 – CSF1R
COL6A3 – SDC4
COL6A3 – CD44
COL6A3 – (ITGA11+ITGB1)
COL6A3 – (ITGA10+ITGB1)
COL6A3 – (ITGA1+ITGB1)
COL6A2 – SDC4
COL6A2 – CD44
COL6A2 – (ITGA11+ITGB1)
COL6A2 – (ITGA10+ITGB1)
COL6A2 – (ITGA1+ITGB1)
COL6A1 – SDC4
COL6A1 – CD44
COL6A1 – (ITGA11+ITGB1)
COL6A1 – (ITGA10+ITGB1)
COL6A1 – (ITGA1+ITGB1)
COL4A2 – SDC4
COL4A2 – CD44
COL4A2 – (ITGA11+ITGB1)
COL4A2 – (ITGA10+ITGB1)
COL4A2 – (ITGA1+ITGB1)
COL4A1 – SDC4
COL4A1 – CD44
COL4A1 – (ITGA11+ITGB1)
COL4A1 – (ITGA10+ITGB1)
COL4A1 – (ITGA1+ITGB1)
COL1A2 – SDC4
COL1A2 – CD44
COL1A2 – (ITGA11+ITGB1)
COL1A2 – (ITGA10+ITGB1)
COL1A2 – (ITGA1+ITGB1)
COL1A1 – SDC4
COL1A1 – CD44
COL1A1 – (ITGA11+ITGB1)
COL1A1 – (ITGA10+ITGB1)
COL1A1 – (ITGA1+ITGB1)
CD99 – PILRA
CD99 – CD99
CD46 – JAG1
CCL2 – CCR2
BMP5 – (BMPR1A+BMPR2)
BMP5 – (BMPR1A+ACVR2A)
APP – CD74
ANXA1 – FPR2
ANXA1 – FPR1
ANGPTL4 – SDC4
ANGPTL4 – SDC3
ANGPTL4 – SDC2
ANGPTL4 – CDH11
ANGPTL2 – TLR4
ANGPTL2 – PIRB
OBs –> B cell
OBs –> OBs
OBs –> Dendritic cell
OBs –> Fibroblast cell
OBs –> Granulocyte
OBs –> Macrophage
OBs –> Myeloid cell
OBs –> Neutrophil
OBs –> T cell
Commun. Prob.
max
min
p–value
p < 0.01

Supplementary Figure S4. Cellchat analysis reveals all the ligand-receptor pathways that have been enriched.
